# A computational model of Alzheimer's disease at the nano, micro, and macroscales

**DOI:** 10.3389/fninf.2024.1348113

**Published:** 2024-03-22

**Authors:** Éléonore Chamberland, Seyedadel Moravveji, Nicolas Doyon, Simon Duchesne

**Affiliations:** ^1^Centre de Recherche CERVO, Institut Universitaire de Santé Mentale de Québec, Québec, QC, Canada; ^2^Département de Mathématiques et de Statistique, Québec, QC, Canada; ^3^Département de Radiologie et Médecine Nucléaire, Université Laval, Québec, QC, Canada; ^4^Centre de Recherche de l'Institut Universitaire en Cardiologie et Pneumologie de Québec, Québec, QC, Canada

**Keywords:** mathematical models, Alzheimer's disease, complex biological systems, personalized approach, APOE, amyloid beta, tau proteins, ordinary differential equations

## Abstract

**Introduction:**

Mathematical models play a crucial role in investigating complex biological systems, enabling a comprehensive understanding of interactions among various components and facilitating *in silico* testing of intervention strategies. Alzheimer's disease (AD) is characterized by multifactorial causes and intricate interactions among biological entities, necessitating a personalized approach due to the lack of effective treatments. Therefore, mathematical models offer promise as indispensable tools in combating AD. However, existing models in this emerging field often suffer from limitations such as inadequate validation or a narrow focus on single proteins or pathways.

**Methods:**

In this paper, we present a multiscale mathematical model that describes the progression of AD through a system of 19 ordinary differential equations. The equations describe the evolution of proteins (nanoscale), cell populations (microscale), and organ-level structures (macroscale) over a 50-year lifespan, as they relate to amyloid and tau accumulation, inflammation, and neuronal death.

**Results:**

Distinguishing our model is a robust foundation in biological principles, ensuring improved justification for the included equations, and rigorous parameter justification derived from published experimental literature.

**Conclusion:**

This model represents an essential initial step toward constructing a predictive framework, which holds significant potential for identifying effective therapeutic targets in the fight against AD.

## 1 Introduction

The dominant etiological theory for Alzheimer's disease (AD) is the amyloid-β (*Aβ*) protein cascade hypothesis, formulated in 1984 (Hardy and Higgins, 1992). It has remained at the center of the conceptualization of the disease ever since, including in diagnostic guidelines (McKhann et al., 2011), research frameworks (Jack et al., 2013), and now clinical care. It has regained new impetus as three *Aβ* antibody therapies are gaining regulatory approval worldwide based on their demonstrating removal of *Aβ* oligomers with an accompanying delay in the cognitive decline of approximately six months (Sims et al., 2023; van Dyck et al., 2023). However, all three “now referred to in the field as disease-modifying therapies” present with amyloid-related imaging abnormalities related to a breakdown in the cerebrovasculature in a high number (35%) of individuals, as well as a large heterogeneity response, with women and non-White populations, in particular, showing less to no benefit (Farrer et al., 1997). Thus, identifying individuals who can safely receive and benefit from these treatments is now a glaring priority.

Further, while these disease-modifying therapies constitute an undeniable advance, their limited efficacy does not eliminate the need to understand the dementia process surrounding *Aβ* accumulation, and attempt to explain discrepant evidence such as its poor relation to cognitive deficits (Mormino et al., 2009; Villain et al., 2012; Haller et al., 2019), its presence at theoretically pathological levels in a large proportion of otherwise cognitively healthy seniors (Katzman et al., 1988; Bennett et al., 2006), and its poor spatial match to the accumulation of tau, neurodegeneration, or brain energy hypometabolism (Jack et al., 2013).

A larger, more integrated perspective is therefore required to understand the relationship between the various abnormalities that are present in AD, such as the aberrant production and accumulation of amyloid and tau proteins, reduced energy metabolism and neurovascular coupling, inflammation surge, synaptic dysfunction and neuronal death, and inevitably, cognitive and behavioral impairment; all the while taking into account genotype differences (e.g., sex and APOE gene) and lifestyle risk factors.

Experimentally, assessing such a global theory is logistically impossible to achieve as the number of variables is substantial. This leads to ethical, logistical, and financial difficulties when studying human patients, namely the lack of causal studies, the long duration of neurodegeneration, and the always incomplete selection of biomarkers in clinical studies due to resource and time constraints. Traditional research further relies on statistical models, a reductionist approach geared toward proving the null hypothesis, and that frequently are used only to test associations between data without providing an understanding of the processes at play.

Alternatively, computational models integrate diverse sources of data into a theoretical framework, able to test the plausibility of working hypotheses and elicit novel ones. Mechanistic computational models are therefore able to capture interactions between abstracted entities at various levels, sidestepping the limitations inherent in human studies, and thus provide a deeper understanding of the interactions between those involved in aging and neurodegeneration. They could be used to uncover potential mechanisms, provide an *in silico* testable environment for treatment response, and hence suggest either novel or more appropriately personalized therapeutic regimens.

Few large-scale theoretical integrative efforts of this kind have been attempted, e.g. Hao and Friedman (2016), Bertsch et al. (2017), Petrella et al. (2019), and Ji et al. (2021). In our recent review of integrative mathematical models of AD, we found near-universal limitations in terms of internal and external validity, reduced scope, or unrealistic disease conceptualizations (Moravveji et al., 2022).

In this work, we propose a multi-entity, multi-scale (protein, cells, and organ-level) mathematical model describing the aging process, to capture the onset and progression of AD, based on and improving upon the one proposed by Hao and Friedman (2016). Our model is described in the following section and covers processes such as extracellular *Aβ* aggregation and clearance, the formation of neurofibrillary tangles (NFTs) from tau proteins, and the activation of microglia. The contributions of our work compared to previous reports include (a) an improved justification, via biological principles, of the qualitative and quantitative relationships between the model's entities; (b) the inclusion of novel entities, especially glycogen synthase kinase 3 (GSK-3) and insulin, to enable the link between brain health and diabetes, a well-identified risk factor for AD (Zhang et al., 2018); (c) a more complex description of the amyloid accumulation process by including different stages for amyloid-β extracellular aggregation (monomers, oligomers, and plaques), enabling the testing of hypotheses related to disease-modifying treatments; (d) the concept of a “reservoir” of non-activated cells, such as non-activated microglia, as well as terms describing the conversion of macrophages and microglia from anti-inflammatory to proinflammatory states; and (e) the inclusion of sex and APOE gene effects on some parameters. We also solved the model over a longer evolution timeframe, describing the effects of aging over 50 years; these results are provided without and with manipulation of some external entities, e.g. insulin concentrations. Finally, we completed a thorough validation of parameter values using published literature; these are listed in Supplementary material A. *In fine*, our model can generate predictions that can be more easily validated with human experimental data, as presented in the Section 4 of our report.

It is important to realize that this represents but a step toward an ever more complex disease model, as with each new evidence the nature of the equations and parameter values are set to change.

## 2 Method

**Figure 1:**
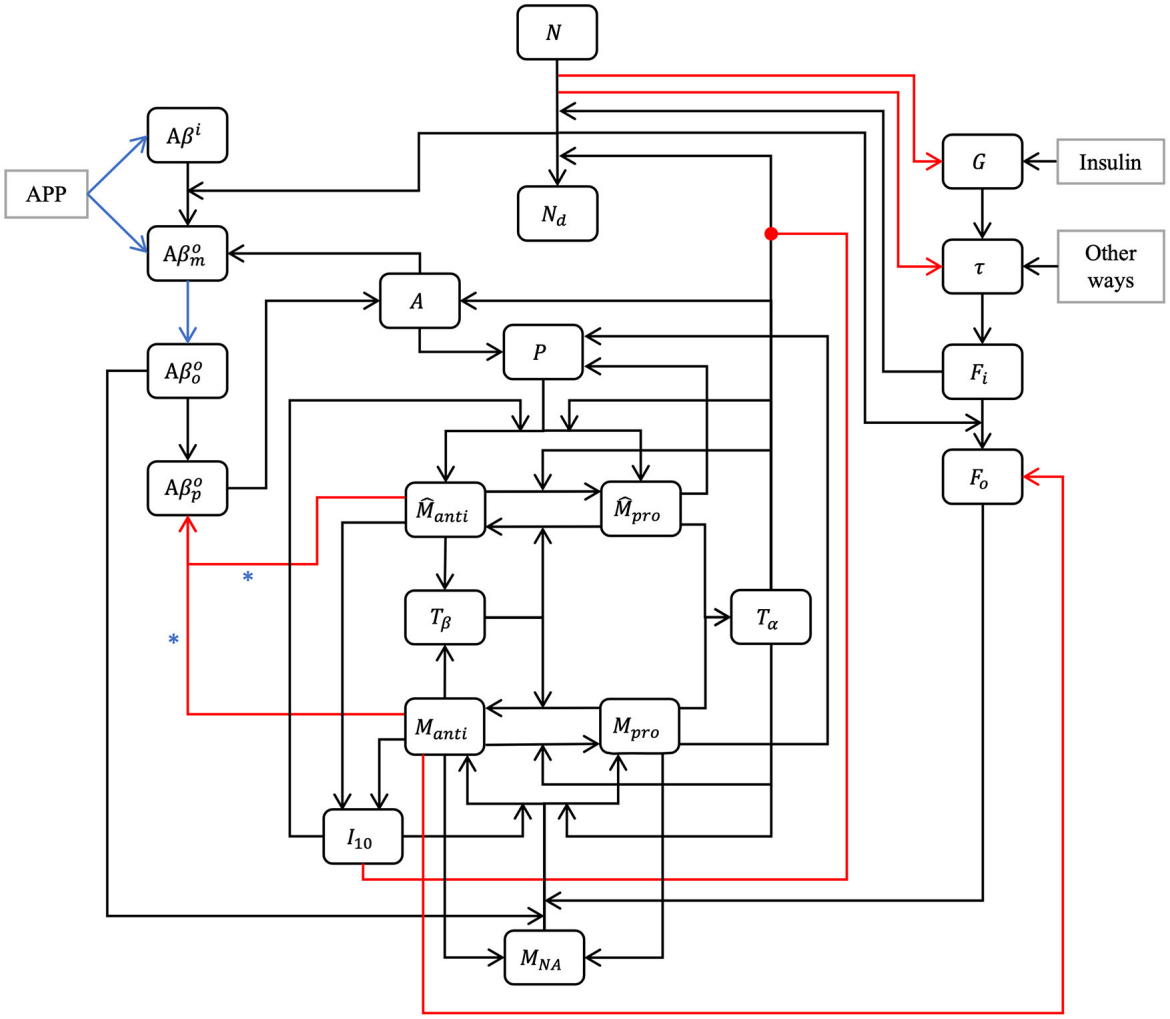
Model schematic. Our hypothesis can be summarized in the model schematic. We propose that the relationships between entities, evolving dynamically through time, is sufficient to explain the decline in neurons, rise of inflammation, and increases in both amyloid plaques and tau tangles that is seen in aging, up to and including Alzheimer's disease, without requiring an external “catalytic” event such as reactive oxygen species production. This is a multi-factorial, multi-parametric viewpoint, quite different from other hypotheses in the literature. Blue arrows and asterisk identify an APOE4-dependent relation. Red arrows identify degradation relations, and red lines ending with a dot is for inhibition.

Our model is composed of 23 ordinary differential or other equations and describes the evolution of brain health entities over 50 years. A schematic illustrating how each entity interact is given in Figure 1. Each entity has been abstracted as a dynamical variable, expressed in either concentration or density (g/ml or g/cm^3^). Table 1 lists all variables of the model.

**Table 1:** Model variables.

*Aβ* ^*i*^	Intracellular Aβ
${A\beta }_{m}^{o}$	Extracellular Aβ monomers
${A\beta }_{o}^{o}$	Extracellular Aβ oligomers
${A\beta }_{m}^{o}$	Extracellular Aβ plaques
*G*	GSK-3
τ	Phosphorylated tau proteins
*F*_*i*_	Intracellular NFT
*F*_*o*_	Extracellular NFT
*N*	Neuron density
*A*	Density of activated astrocytes
*M*_*NA*_	Non activated microglia
*M*_*pro*_	Proinflammatory microglia
*M*_*anti*_	Anti-inflammatory microglia
${\hat{M}}_{pro}$	Proinflammatory macrophages
*T*_β_	TGF-β
*T*_α_	TNF-α
*I*_10_	IL-10
*P*	MCP-1

### 2.1 Impact of sex

Around 60% of Americans suffering from AD are women (Rajan et al., 2021). This has been proposed to be related to the action of hormones, estrogens in particular. Some authors claim a link between menopause and dementia (Mielke, 2018). Generally speaking, women experience a more rapid cognitive decline than men. It is possible that this is due to the fact that women receive an AD diagnosis later in life than men (Ferretti et al., 2018). According to many studies, there are no differences with respect to the Aβ charge between men and women (Ferretti et al., 2018). This is however controversial as other studies demonstrate sex-related differences, such as Carroll et al. (2010) and Yang J.-T. et al. (2018). Regarding neurofibrillary tangles and hyperphosphorylated tau proteins, the majority of post-mortem studies of AD patients did not reveal an impact of sex (Ferretti et al., 2018). However, some studies suggested that there is a greater accumulation of tau proteins and NFT in women than in men (Barnes et al., 2005; Yang J.-T. et al., 2018). Studies using large-scale datasets such as *Alzheimer's Disease Neuroimaging Initiative* (ADNI) and *Minimal Interval Resonance Imaging in Alzheimer's Disease* (MIRIAD) determined that atrophy rates are 1%–1.5% faster in women suffering from mild cognitive decline or AD than in men with similar conditions (Ferretti et al., 2018), which would be an indicator of a greater neural loss and more important tau pathology. We elected to take sex into account in our model by including its effect in various equations via the variable *S* (*S* = 0 for women and *S* = 1 for men); capturing sex-related differences in the densities of neurons, astrocytes, and microglia (Pakkenberg and Gundersen, 1997; Pelvig et al., 2008); as well as using different functions and values for cerebral insulin based on the findings of Bryhni et al. (2010). The normal GSK-3 concentration is also sex-dependent according to Knight et al. (2021).

### 2.2 Impact of APOE4

One of the most important genetic risks for AD is the presence of one or two copies of the ε4 allele of the APOE gene (APOE4). About 15%–25% of individuals bear a copy of APOE4 and 2%–3% have two copies of the gene: the ε3 allele is the most common and is not associated with an increased risk of AD. The ε2 allele is relatively rare and could offer protection against AD (Hsiung and Dessa Sadovnick, 2007; Bryant, 2021). People with APOE4 have a greater accumulation of Aβ (Roher et al., 2009; Kanekiyo et al., 2014). In our model, we supposed that APOE4 leads to greater intracellular Aβ production. We also assumed that APOE4 leads to a greater rate of extracellular Aβ production (Roher et al., 2009). It has been observed by Hashimoto et al. (2012) that the level of Aβ oligomers in persons suffering from AD and having APOE4 is 2.7 times greater than in people with APOE3. In our model, we considered that the aggregation rate of Aβ into oligomers is higher in people with APOE4. We discriminated between APOE4 status by defining a variable, *AP*, for the presence of APOE4 (*AP* = 1 when the allele ε4 is expressed and *AP* = 0 otherwise). We considered that APOE4 has no impact on the aggregation rate of Aβ oligomers into plaques (Garai et al., 2014; Jäkel et al., 2020). In our model, we rather chose as a mechanism of action that clearance of Aβ plaques was less efficient in persons with APOE4 (Kanekiyo et al., 2014; Hansen et al., 2018).

### 2.3 Neuronal populations

From Pelvig et al. (2008), we extracted the values for the number and proportion of cells of each type (see Table 2).

**Table 2:** Populations (in billions) and proportions (in %) of brain cells, based on Pelvig et al. (2008).

**Cell type**	**Women**		**Men**	
	**Number of cells (**×10^9^**)**	**Percentage (%)**	**Number of cells (**×10^9^**)**	**Percentage (%)**
Neurons	21.4	43.4	26.3	41.3
Oligodendrocytes	21.0	42.6	28.8	44.2
Astrocytes	4.8	9.7	7.8	12.0
Microglaes	1.8	3.7	2.0	3.1
Other glial cells	0.3	0.6	0.3	0.5
Total	49.3	100	65.2	100

Knowing that the brain density, ρ_br_, is 1.03 g/cm3 (National Institute of Standards and Technology, 2017), we can compute the density of neurons and astrocytes in the female (*F*) and male (*M*) brain (see Equations 1–4).


(1) $$N_{0}^{F}\;=\eta_{N^{F}}\times \rho_{\text{br}}=\frac{43.4}{100}\times 1.03=\;0.45\;{\text{g/cm}}^{3},$$

(2) $$N_{0}^{M}\;=\eta_{N^{M}}\times \rho_{\text{br}}=\frac{41.3}{100}\times 1.03=\;0.42\;{\text{g/cm}}^{3},$$

(3) $$A_{0}^{F}\;=\eta_{A^{F}}\times \rho_{\text{br}}=\frac{9.7}{100}\times 1.03=\;0.10\;{\text{g/cm}}^{3},$$

(4) $$A_{0}^{M}\;=\eta_{A^{M}}\times \rho_{\text{br}}=\frac{12.0}{100}\times 1.03=\;0.12\;{\text{g/cm}}^{3},$$

where η_*X*_ is the percentage of brain cells corresponding to the population *X*, with *X* equal to *N* for neurons or *A* for astrocytes.

### 2.4 Equations for amyloid beta

AD is characterized by Aβ plaque deposits. These plaques are produced by the amyloid precursor protein after it has been sequentially cleaved by β- and γ-secretase enzymes. This process leads to the creation of two types of Aβ: Aβ40 and Aβ42. While Aβ42 only accounts for 10% of the total quantity of Aβ, it is the most toxic form due to its hydrophobicity, aggregation, and a larger potential for fibrillation (Hohsfield and Humpel, 2015). The agglomeration process of Aβ is still little understood. Some studies suggest that oligomers are only a transitory form in fibril formation while other studies propose that these different species emerge from independent pathways (Cohen et al., 2013). It is not clear if oligomer aggregation occurs through successive monomer addition or the concatenation of smaller oligomers. For tetramers, it appears that the one-at-a-time aggregation of monomer is more frequent than the concatenation of two dimers (Man et al., 2019). However, the opposite result has also been proposed (Barz et al., 2018). Either way, small oligomers aggregate to form protofibrils, which can become fibrils, which can in turn form plaques. According to this evidence, we will only consider Aβ42 and divide aggregation into four phases: intracellular monomers, and extracellular monomers, oligomers, and plaques. We assume that monomers cleaved by γ-secretase aggregate into oligomers.

#### 2.4.1 Equation for intracellular amyloid beta monomers

The equation for monomeric Aβ is:


(5) $$\begin{array}{c} \frac{dA\beta^{i}}{dt}=\lambda_{{A\beta }^{i}}\left(1+AP\cdot \delta_{APi}\right)\frac{N}{N_{0}}-d_{{A\beta }^{i}}{A\beta }^{i}-\frac{{A\beta }^{i}}{N}|\frac{dN}{dt}|. \end{array}$$

The first term in Equation (5) represents the creation of intracellular Aβ from an amyloid precursor protein, where $\lambda_{{A\beta }^{i}}$ is the reaction rate influenced by the presence of APOE4.

The second term, $-d_{{A\beta }^{i}}{A\beta }^{i}$, describes the degradation of intracellular Aβ, with $d_{{A\beta }^{i}}$ representing the rate of degradation.

The last term in Equation (5) accounts for the externalization of intracellular monomers when neurons undergo cell death. This term captures the transition of intracellular Aβ to extracellular Aβ and is proportional to the rate of neuronal loss, denoted by $\frac{dN}{dt}$.

#### 2.4.2 Equation for extracellular amyloid beta monomers

For extracellular Aβ, we considered two separate aggregation processes: the transition from monomers to oligomers and subsequently the transition from oligomers to plaques.

The equation describing the evolution of extracellular Aβ monomers is given by


(6) $$\begin{array}{l} \frac{dA\beta_{m}^{o}}{dt}=\frac{A\beta^{i}}{N}|\frac{dN}{dt}|+\lambda_{A\beta_{m}^{o}}(1+AP\cdot \delta_{APm})\frac{N}{N_{0}}+\lambda_{AA\beta_{m}^{o}}\frac{A}{A_{0}}\\ \;\;\;\;\;\;\;\;\;\;\;\;\;-\kappa_{A\beta_{m}^{o}A\beta_{o}^{o}}(1+AP\cdot \delta_{APmo}){\left(A\beta_{m}^{o}\right)}^{2}-d_{A\beta_{m}^{o}}A\beta_{m}^{o}. \end{array}$$

The first term of Equation 6 represents the transition from intracellular to extracellular amyloid, while the second term represents the creation of extracellular Aβ monomers from the amyloid precursor protein. The rate of production is assumed to be proportional to neuron density. The third term accounts for the production of extracellular Aβ monomers by activated astrocytes, which produce less Aβ compared to neurons. The rate of production is denoted by $\lambda_{A{A\beta }_{m}^{o}}$, it is influenced by the presence of APOE4 and is proportional to astrocyte activity.

The aggregation of monomers into oligomers is described by the fourth term, which is influenced by the presence of APOE4. The rate of aggregation is represented by $\kappa_{{A\beta }_{m}^{o}{A\beta }_{o}^{o}}$, and it reflects the propensity of Aβ monomers to form oligomers. Garai and Frieden (2013) identified the formation of dimers as a critical step in the transition from monomers to oligomers. Although our equation focuses on dimer formation (hence the squared term), for simplicity and due to data limitations, we consider the rate as a representative step denoted by $\kappa_{{A\beta }_{m}^{o}{A\beta }_{o}^{o}}$. Lastly, the equation accounts for the degradation of extracellular Aβ monomers through various processes, including degradation by microglia and self-degradation. The rate of degradation, represented by $d_{{A\beta }_{m}^{o}}$, reflects the overall degradation rate of extracellular Aβ monomers.

#### 2.4.3 Equation for extracellular amyloid beta oligomers


(7) $$\begin{array}{l} \frac{dA\beta_{o}^{o}}{dt}=\kappa_{A\beta_{m}^{o}A\beta_{o}^{o}}(1+AP\cdot \delta_{APmo}){\left(A\beta_{m}^{o}\right)}^{2}-\kappa_{A\beta_{o}^{o}A\beta_{p}^{o}}{\left(A\beta_{o}^{o}\right)}^{2}\\ \;\;\;\;\;\;\;\;\;\;\;\;\;\;-d_{A\beta_{o}^{o}}A\beta_{o}^{o}. \end{array}$$

The Equation 7 consists of three terms. The first one accounts for the aggregation of monomers into oligomers; the second describes the aggregation of oligomers into *Aβ* plaques, with $\kappa_{{A\beta }_{o}^{o}{A\beta }_{p}^{o}}$ representing the aggregation rate. Finally, the last term captures the degradation of oligomers through various processes, including microglia-mediated degradation and self-degradation.

#### 2.4.4 Equation for extracellular amyloid beta plaques


(8) $$\begin{array}{l} \frac{dA\beta_{p}^{o}}{dt}=\;\kappa_{A\beta_{o}^{o}A\beta_{p}^{o}}{\left(A\beta_{o}^{o}\right)}^{2}\\ \;\;\;\;\;\;\;\;\;\;\;\;\;\;\;\;-(d_{M_{anti}A\beta_{p}^{o}}M_{anti}+d_{{\hat{M}}_{anti}A\beta_{p}^{o}}{\hat{M}}_{anti})\\ \;\;\;\;\;\;\;\;\;\;\;\;\;\;\;\;(1+AP\cdot \delta_{APdp})\frac{A\beta_{p}^{o}}{A\beta_{p}^{o}+K_{A\beta_{p}^{o}}}. \end{array}$$

The first term of Equation 8 represents an oligomer-to-plaque conversion while the second one describes plaque degradation by anti-inflammatory microglia and macrophages.

Activated macrophages and microglia can eliminate Aβ plaques (Lee and Landreth, 2010). Macrophages are more efficient at this task than microglia (Lai and McLaurin, 2012; Thériault et al., 2015). Also anti-inflammatory microglia and macrophages cleave Aβ more efficiently than their proinflammatory counterparts (Tang and Le, 2016; Wang et al., 2021). For this reason, we will neglect plaque degradation by proinflammatory cells.

The constants $d_{M_{anti}{A\beta }_{p}^{o}}$ and $d_{{\hat{M}}_{anti}{A\beta }_{p}^{o}}$ denote the maximum degradation rates of Aβ plaques by microglia and macrophages, respectively. Lastly, the constant $K_{{A\beta }_{p}^{o}}$ represents the half-saturation constant for plaque degradation by microglia and macrophages.

### 2.5 Equation for glycogen synthase kinase 3

There are two isoenzymes of GSK-3, GSK-3α and GSK-3β, both able to hyperphosphorylate tau proteins and are putatively involved in AD. However, GSK-3β is more dysregulated in AD (Hooper et al., 2008). For this reason, our model will only consider that isoenzyme. In turn, the action of GSK-3 is modulated by insulin concentration. When the insulin concentration is normal, GSK-3 is inhibited, but when it is smaller, the activity of GSK-3 is increased (Cross et al., 1995; Ghasemi et al., 2013; Yang L. et al., 2018).


(9) $$\begin{array}{c} \frac{dG}{dt}=\lambda_{\text{Ins}G}\frac{{\text{Ins}}_{0}}{\text{Ins}\left(t\right)}\frac{N}{N_{0}}-d_{G}G-\frac{G}{N}|\frac{dN}{dt}|. \end{array}$$

In this context, in Equation 9, λ_Ins*G*_ represents the insulin-influenced rate of GSK-3 creation, Ins(*t*) denotes the insulin concentration as a function of age, while Ins_0_ represents the normal concentration of brain insulin. The second term describes GSK-3 degradation; the constant *d*_*G*_ refers to the rate of this decrease/degradation.

### 2.6 Equation for phosphorylated/hyperphosphorylated tau proteins

Another important factor in AD onset and progression is the presence of hyperphosphorylated tau proteins (τ) in the central nervous system, mainly in neurons. Tau proteins are phosphorylated and hyperphosphorylated by many processes including glycogen synthase kinase 3 (GSK-3) (Domínguez et al., 2012). Tau proteins in AD patients are three to four times more phosphorylated than in age-matched individuals without cognitive difficulties (Gong and Iqbal, 2008). The equation for τ is as follows:


(10) $$\begin{array}{c} \frac{d\tau }{dt}=\lambda_{\tau }\frac{N}{N_{0}}+\lambda_{G\tau }\frac{G}{G_{0}}-\kappa_{\tau F_{i}}{\left(\tau \right)}^{2}\frac{N}{N_{0}}-\frac{\tau }{N}|\frac{dN}{dt}|-d_{\tau }\tau . \end{array}$$

The first term accounts for non-GSK-3-mediated hyperphosphorylation of tau proteins. The constant λ_τ_ represents the initial rate of this hyperphosphorylation. The second term corresponds to GSK-3-mediated hyperphosphorylation. Deviations in GSK-3 concentration from the normal level *G*_0_ result in either increased or decreased tau protein hyperphosphorylation compared to the normal rate (λ_*Gτ*_). The third term corresponds to the transformation of tau proteins into neurofibrillary tangles. The exponent 2 translates the fact that at least two tau proteins are necessary to form a neurofibrillary tangle. When neurons die, tau proteins are released from the intracellular space into the extracellular environment and are subsequently eliminated. This process is captured by the term $-\frac{\tau }{N}|\frac{dN}{dt}|$ in Equation (10). The last term accounts for the degradation and dephosphorylation of phosphorylated/hyperphosphorylated tau proteins at a rate of *d*_τ_.

### 2.7 Equations for NFTs

Hyperphosphorylated tau proteins aggregate to form neurofibrillary tangles (NFTs), causing the destruction of microtubules, which in turn blocks the neuron transport system and affects synaptic transmission (Hao and Friedman, 2016; National Institute on Aging, 2017). The presence of intracellular NFTs leads to neural death. The equation for intracellular NFTs is as follows:


(11) $$\begin{array}{c} \frac{dF_{i}}{dt}=\kappa_{\tau F_{i}}{\left(\tau \right)}^{2}\frac{N}{N_{0}}-d_{F_{i}}F_{i}-\frac{F_{i}}{N}|\frac{dN}{dt}|. \end{array}$$

The first term captures the aggregation process of hyperphosphorylated tau proteins, resulting in the formation of intracellular NFTs. The second term accounts for the degradation and elimination of these intracellular NFTs at a rate of *d*_*F*_*i*__. Finally, the last term represents the release of intracellular NFTs into the extracellular space upon neuronal death.

The evolution of extracellular NFTs is described by the following equation


(12) $$\begin{array}{c} \frac{dF_{o}}{dt}=\frac{F_{i}}{N}|\frac{dN}{dt}|-\kappa_{MF_{o}}\frac{M_{anti}}{M_{anti}+K_{M_{anti}}}F_{o}-d_{F_{o}}F_{o}. \end{array}$$

The first term in this equation, which is the same as the last term in Equation (11), represents the release of extracellular neurofibrillary tangles upon neuronal death. When neurons die, intracellular NFTs transform into “ghost tangles” that undergo minimal degradation, unless microglia or astrocytes act (Kril et al., 2002; Zilka et al., 2012; Moloney et al., 2021). The degradation of extracellular NFTs by microglia, specifically anti-inflammatory microglia, is described by the second term in Equation (12). The last term accounts for the degradation of extracellular NFTs resulting from other factors such as astrocytes and self-degradation.

### 2.8 Equation for the density of neurons

The evolution of neuronal density is described by the following equation:


(13) $$\begin{array}{l} \frac{dN}{dt}=-d_{F_{i}N}\frac{1}{1+\exp(-n\cdot \frac{F_{i}-K_{F_{i}}}{K_{F_{i}}})}N\\ \;-d_{T_{\alpha }N}\frac{T_{\alpha }}{T_{\alpha }+K_{T_{\alpha }}}\frac{1}{1+I_{10}/K_{I_{10}}}N, \end{array}$$

where *n* is the sigmoid coefficient determining its slope. The first term in Equation (13) represents neural death caused by intracellular NFTs (*F*_*i*_), where *d*_*F*_*i*__*N* denotes the rate of neural death attributed to *F*_*i*_, and *K*_*F*_*i*__ represents the concentration of *F*_*i*_ at which this rate is half-maximal.

Neural death is also related to the presence of proinflammatory cytokines, such as tumor necrosis factor-alpha (TNF-α). However, the pathway leading to this apoptosis is unclear even though many theories have been proposed (Takeuchi et al., 2006; Park and Bowers, 2010; Neniskyte et al., 2014; Wang et al., 2015). For the sake of simplicity, we will assume that TNF-α directly causes neural death. It is known that neural death caused by TNF-α is inhibited by the anti-inflammatory cytokines, in particular the interleukin (IL)-10 (Porro et al., 2020). This relationship is captured by the second term in Equation (13), wherein *d*_*T*_α_*N*_ denotes the maximum rate of neural death caused by proinflammatory cytokines, *K*_*T*_α__ represents the concentration of TNF-α at which the rate of neural death is half-maximal, and *K*_*I*_10__ corresponds to the concentration of IL-10 at which this rate is reduced by half. This term corresponds to the Michaelis–Menten equation for reaction velocity with a non-competitive inhibitor.

### 2.9 Equation for the density of activated astrocytes

Astrocytes are activated by TNF-α and Aβ plaques (Nagele et al., 2004; Morales et al., 2014; Liddelow et al., 2017). Activated astrocytes produce Aβ monomers but in smaller quantity than neurons (Blasko et al., 2000; Zhao et al., 2011). Our equation for activated astrocytes is as follows:


(14) $$\begin{array}{c} \frac{dA}{dt}=\kappa_{T_{\alpha }A}T_{\alpha }\left(A_{\text{max}}-A\right)+\kappa_{{A\beta }_{p}^{o}A}{A\beta }_{p}^{o}\left(A_{\text{max}}-A\right)-d_{A}A. \end{array}$$

The first term describes the activation of astrocytes by TNF-α and the second one describes activation by Aβ plaques. We multiply the production terms by the factor (*A*_max_ − *A*), where *A*_max_ represents the maximum density of activated astrocytes. We assume that all astrocytes can become activated. The parameters κ_*T*_α_*A*_ and $\kappa_{{A\beta }_{p}^{o}A}$ correspond to the rates of astrocyte activation by TNF-α and Aβ plaques, respectively.

The last term in Equation (14) represents the deactivation and death of activated astrocytes. According to Sofroniew (2020, figure 2), astrocytes return to their initial state if the issue is resolved and can transition to a chronic state if the problem persists. To capture this complexity accurately, the model for astrocytes would need to be more sophisticated. However, for the sake of simplicity, we assume a constant death rate *d*_*A*_.

### 2.10 Equations for microglia

#### 2.10.1 Resting microglia

Microglia are resident macrophages of the central nervous system. They eliminate pathogens, dying cells, and detritus. They are also involved in tissue repair (Orihuela et al., 2016). In our model, we consider microglia as either resting (non-activated) or activated and, if activated, in one of the two states: pro or anti-inflammatory. Microglia are activated by Aβ oligomers (Michelucci et al., 2009; Tang and Le, 2016) and extracellular NFTs (Maccioni et al., 2010; Ohm et al., 2021). We assume that the sum of activated and non-activated microglia is approximately constant (from figure 3 of Pelvig et al., 2008). Therefore, when proinflammatory or anti-inflammatory microglia die or are deactivated, they are replaced by resting microglia. This occurs at rates *d*_*M*_*pro*__ and *d*_*M*_*anti*__, respectively. The rate of activation of microglia is a function of extracellular NFT concentration (*F*_*o*_) and of the concentration of extracellular Aβ oligomers ($A\beta_{o}^{o}$). The evolution of non-activated microglia is described by the Equation (15).


(15) $$\begin{array}{c} \frac{dM_{NA}}{dt}=d_{M_{pro}}M_{pro}+d_{M_{anti}}M_{anti}-M_{activ}, \end{array}$$

where *M*_*activ*_ represents the variation of microglia becoming active and is given by Equation (16).


(16) $$\begin{array}{c} M_{activ}=\kappa_{F_{o}M}\frac{F_{o}}{F_{o}+K_{F_{o}}}M_{NA}+\kappa_{{A\beta }_{o}^{o}M}\frac{{A\beta }_{o}^{o}}{{A\beta }_{o}^{o}+K_{{A\beta }_{o}^{o}}}M_{NA}. \end{array}$$

#### 2.10.2 Activated microglia

We use a discreet model with microglia being either proinflammatory or anti-inflammatory, even though polarization is a continuum between these two extremes. The proinflammatory polarization is triggered by the signaling of proinflammatory cytokines mainly gamma interferon (IFN-γ) and TNF-α as well as lipopolysaccharides (LPS) (Martinez and Gordon, 2014; Orihuela et al., 2016; Wang et al., 2021). We consider TNF-α and assume that its concentration is proportional to the concentration of the other cytokines. Analogously, the anti-inflammatory polarizing is triggered by the signaling of anti-inflammatory cytokines such as IL-4, IL-10, IL-13, and transforming growth factor beta (TGF-β) (Martinez and Gordon, 2014; Orihuela et al., 2016; Wang et al., 2021). We will use IL-10 and assume that its concentration is proportional to the concentration of the other cytokines.

Furthermore, under the signaling of TGF-β, proinflammatory microglia can change their polarization and become anti-inflammatory (Song et al., 2022). We let κ_*T*_β_*M*_*pro*__ stand for the maximal rate of conversion and *K*_*T*_β_*M*_ stand for the TGF-β concentration at which this conversion rate is half maximal.

Moreover, under the signaling of TNF-α, anti-inflammatory microglia can be converted to proinflammatory (Tang and Le, 2016). We let κ_*T*_α_*M*_*anti*__ stand for the maximal rate of this conversion and *K*_*T*_α_*M*_ stand for the concentration of TNF-α for which this conversion rate is half maximal.

We obtain the following equations for proinflammatory, *M*_*pro*_, and anti-inflammatory, *M*_*anti*_, activated microglia (see Equations 17 and 18).


(17) $$\begin{array}{l} \frac{dM_{pro}}{dt}=\frac{\beta \varepsilon_{T_{\alpha }}}{\beta \varepsilon_{T_{\alpha }}+\varepsilon_{I_{10}}}M_{activ}-\kappa_{T_{\beta }M_{pro}}\frac{T_{\beta }}{T_{\beta }+K_{T_{\beta }M}}M_{pro}\\ \;+\kappa_{T_{\alpha }M_{anti}}\frac{T_{\alpha }}{T_{\alpha }+K_{T_{\alpha }M}}M_{anti}-d_{M_{pro}}M_{pro}, \end{array}$$

(18) $$\begin{array}{l} \frac{dM_{anti}}{dt}=\frac{\varepsilon_{I_{10}}}{\beta \varepsilon_{T_{\alpha }}+\varepsilon_{I_{10}}}M_{activ}+\kappa_{T_{\beta }M_{pro}}\frac{T_{\beta }}{T_{\beta }+K_{T_{\beta }M}}M_{pro}\\ \;-\kappa_{T_{\alpha }M_{anti}}\frac{T_{\alpha }}{T_{\alpha }+K_{T_{\alpha }M}}M_{anti}-d_{M_{anti}}M_{anti}, \end{array}$$

where β is the environmental ratio of proinflammatory over anti-inflammatory as determined by the relative strength of TNF-α and IL-10, besides ε_*T*_α__ and ε_*I*_10__ are defined in Equation (19).


(19) $$\begin{array}{c} \varepsilon_{T_{\alpha }}=\frac{T_{\alpha }}{T_{\alpha }+K_{T_{\alpha }Act}},\;\text{and }\varepsilon_{I_{10}}=\frac{I_{10}}{I_{10}+K_{I_{10}Act}}, \end{array}$$

where *K*_*T*_α_*Act*_ is the half saturation constant of TNF-α for proinflammatory polarization and *K*_*I*_10_*Act*_ is the half saturation constant of IL-10 for anti-inflammatory polarization.

### 2.11 Equations for activated macrophages

Macrophages enter the brain via blood vessels under the signaling of the monocyte chemotactic protein (MCP)-1 (Deshmane et al., 2009; Thériault et al., 2015; Lee et al., 2018). The brain macrophages, which are not microglia, originate from blood vessels. The speed with which they are imported into the brain depends on the concentration of MCP-1 (Deshmane et al., 2009). We assume that macrophages can reach a maximal concentration of ${\hat{M}}_{max}$. We let $\kappa_{P\hat{M}}$ stand for the maximal rate of macrophages import in the brain under MCP-1 signaling and *K*_*P*_ stand for the concentration of MCP-1 for which this rate is half maximal.

Macrophages can be activated, and when activated, they are polarized. We use two discreet polarization states, proinflammatory and anti-inflammatory. The role of proinflammatory macrophages (type M1) is to destroy pathogens and to provide antigens to the immune system. On the other hand, anti-inflammatory macrophages (type M2) eliminate inflammation and repair damaged tissues (Redka et al., 2018). The proinflammatory polarizing of macrophages is due to the signaling of proinflammatory cytokines such as gamma interferon (IFN-γ) and TNF-α as well as lipopolysaccharides (Martinez and Gordon, 2014; Orihuela et al., 2016; Wang et al., 2021). Anti-inflammatory polarizing is triggered by anti-inflammatory cytokines, e.g., IL-4, IL-10, IL-13, and TGF-β (Martinez and Gordon, 2014; Orihuela et al., 2016; Wang et al., 2021). For simplicity, in our model, polarization will be a function of only one cytokine of each polarization: IL-10 and TNF-α.

Proinflammatory macrophages, as microglia, can become anti-inflammatory under signaling of TGF-β (Orihuela et al., 2016; Song et al., 2022). We let $\kappa_{T_{\beta }{\hat{M}}_{pro}}$ stand for the maximal rate of this conversion and $K_{T_{\beta }\hat{M}}$ stand for the TGF-β concentration for which this rate of conversion is half maximal.

The inverse conversion is also possible and is caused by TNF-α (Tang and Le, 2016). We let $\kappa_{T_{\alpha }{\hat{M}}_{anti}}$ stand for the maximal rate of this conversion and $K_{T_{\alpha }\hat{M}}$ stand for the TNF-α concentration for which this rate of conversion is half maximal.

Finally, macrophages can die or be deactivated at rates $d_{{\hat{M}}_{pro}}$ and $d_{{\hat{M}}_{anti}}$ for pro and anti-inflammatory macrophages, respectively.

We obtain the following Equations (20, 21) to describe the concentration of proinflammatory and anti-inflammatory activated macrophages:


(20) $$\begin{array}{l} \frac{d{\hat{M}}_{pro}}{dt}=\;\kappa_{P\hat{M}}\frac{P}{P+K_{P}}\left({\hat{M}}_{max}-({\hat{M}}_{pro}+{\hat{M}}_{anti})\right)\frac{\beta \varepsilon_{T_{\alpha }}}{\beta \varepsilon_{T_{\alpha }}+\varepsilon_{I_{10}}}\\ \;-\kappa_{T_{\beta }{\hat{M}}_{pro}}\frac{T_{\beta }}{T_{\beta }+K_{T_{\beta }\hat{M}}}{\hat{M}}_{pro}+\kappa_{T_{\alpha }{\hat{M}}_{anti}}\frac{T_{\alpha }}{T_{\alpha }+K_{T_{\alpha }\hat{M}}}{\hat{M}}_{anti}\\ \;-d_{{\hat{M}}_{pro}}{\hat{M}}_{pro}, \end{array}$$

(21) $$\begin{array}{l} \frac{d{\hat{M}}_{anti}}{dt}=\;\kappa_{P\hat{M}}\frac{P}{P+K_{P}}\left({\hat{M}}_{max}-({\hat{M}}_{pro}+{\hat{M}}_{anti})\right)\frac{\varepsilon_{I_{10}}}{\beta \varepsilon_{T_{\alpha }}+\varepsilon_{I_{10}}}\\ \;+\kappa_{T_{\beta }{\hat{M}}_{pro}}\frac{T_{\beta }}{T_{\beta }+K_{T_{\beta }\hat{M}}}{\hat{M}}_{pro}-\kappa_{T_{\alpha }{\hat{M}}_{anti}}\frac{T_{\alpha }}{T_{\alpha }+K_{T_{\alpha }\hat{M}}}{\hat{M}}_{anti}\\ \;-d_{{\hat{M}}_{anti}}{\hat{M}}_{anti}. \end{array}$$

We recognize here the form of the equations for microglia. The constant β still stands for the polarization ratio and ε_*T*_α__ as well as ε_*I*_10__ are defined as in the section for the equations for activated microglia (Equation 19).

### 2.12 Equations for cytokines and chemokines

Our model also describes the evolution of relevant cytokines and chemokines including TGF-β, IL-10, TNF-α, and MCP-1.

#### 2.12.1 Transforming growth factor beta

Anti-inflammatory microglia and macrophages produce anti-inflammatory cytokines IL-10, IL-13, IL-4, and TGF-β which allows tissue remodeling and reparation as well as angiogenesis (Wang et al., 2015; Orihuela et al., 2016; Tang and Le, 2016). TGF-β is produced by anti-inflammatory microglia and macrophages at respective rates of κ_*M*_*anti*_*T*_β__ and $\kappa_{{\hat{M}}_{anti}T_{\beta }}$. It is degraded at a rate of *d*_*T*_β__. We thus have the Equation (22):


(22) $$\begin{array}{c} \frac{dT_{\beta }}{dt}=\kappa_{M_{anti}T_{\beta }}M_{anti}+\kappa_{{\hat{M}}_{anti}T_{\beta }}{\hat{M}}_{anti}-d_{T_{\beta }}T_{\beta }. \end{array}$$

#### 2.12.2 Interleukin 10

Interleukin 10 (IL-10) is a protective factor mitigating neural death due to TNF-α (Porro et al., 2020). IL-10 is produced by anti-inflammatory macrophages and microglia (Wang et al., 2015; Orihuela et al., 2016; Tang and Le, 2016) at respective rates of κ_*M*_*anti*_*I*_10__ and $\kappa_{{\hat{M}}_{anti}I_{10}}$. It is degraded at a rate *d*_*I*_10__. The equation describing the evolution of IL-10 is thus given by the Equation (23):


(23) $$\begin{array}{c} \frac{dI_{10}}{dt}=\kappa_{M_{anti}I_{10}}M_{anti}+\kappa_{{\hat{M}}_{anti}I_{10}}{\hat{M}}_{anti}-d_{I_{10}}I_{10}. \end{array}$$

#### 2.12.3 Tumor necrosis factor-alpha

Proinflammatory microglia and macrophages are neurotoxic as they produce proinflammatory cytokines such as TNF-α, IL-6, IL-12, and IL-1β (Morales et al., 2014; Wang et al., 2015; Liddelow et al., 2017). TNF-α is produced by proinflammatory microglia and macrophages at respective rates of κ_*M*_*pro*_*T*_α__ and $\kappa_{{\hat{M}}_{pro}T_{\alpha }}$. The equation describing the evolution of *T*_α_ is thus given by the Equation (24):


(24) $$\begin{array}{c} \frac{dT_{\alpha }}{dt}=\kappa_{M_{pro}T_{\alpha }}M_{pro}+\kappa_{{\hat{M}}_{pro}T_{\alpha }}{\hat{M}}_{pro}-d_{T_{\alpha }}T_{\alpha }, \end{array}$$

where *d*_*T*_α__ is the degradation rate of TNF-α.

#### 2.12.4 Monocyte chemoattractant protein-1

Monocytes/macrophages are the main sources of monocyte chemoattractant protein-1 (MCP-1) (Lee et al., 2018). In our model, we consider that MCP-1 is produced by proinflammatory macrophages and microglia (Orihuela et al., 2016; Bardi et al., 2018), as well as by activated astrocytes (Lee et al., 2018). We thus have the Equation (25):


(25) $$\begin{array}{c} \frac{dP}{dt}=\kappa_{M_{pro}P}M_{pro}+\kappa_{{\hat{M}}_{pro}P}{\hat{M}}_{pro}+\kappa_{AP}A-d_{P}P, \end{array}$$

where κ_*M*_*pro*_*P*_ and $\kappa_{{\hat{M}}_{pro}P}$ are the maximal production rate of MCP-1 by proinflammatory microglia and macrophages respectively. The constant κ_*AP*_ denotes the production rate by activated astrocyte and *d*_*P*_ is the rate of degradation by MCP-1.

### 2.13 Numerical implementation

Our model consists of a system of ordinary differential equations (ODEs) which can be written in the normal form ${\vec{y}}^{\prime }\left(t\right)=\vec{f}\left(\vec{y}\left(t\right),t\right)$, with initial conditions $\vec{y}\left(0\right)=\vec{y_{0}}$.

The ODE system is non-autonomous due to explicit time dependence in the function $\vec{f}$. For example, we assumed that insulin concentration varies with age. The model was implemented in Python and the equations were solved using the integrate.solve_ivp function of SciPy package (Virtanen et al., 2020), with method “BDF” (based on a backward differentiation formula). The different timescales present in the model are the telltale signs of a stiff system of ODEs. We thus used a resolution method adapted for stiff equations. To get a good precision, we defined the parameter “atol” and “rtol” of the function. We took rtol = 1 × 10^−5^ and atol = 1 × 10^−22^, for a precision of five digits after the decimal point.

## 3 Results

We first present results obtained with our model as detailed in the preceding section, without modifying the input parameters. We will then explore the impact of modifying the insulin concentration function and then of reduced values for the activation rates of pro and anti-inflammatory microglia, both as proofs of concept of the usefulness of such approaches at testing risk factors and treatment hypotheses.

### 3.1 Standard model

**Figure 2:**
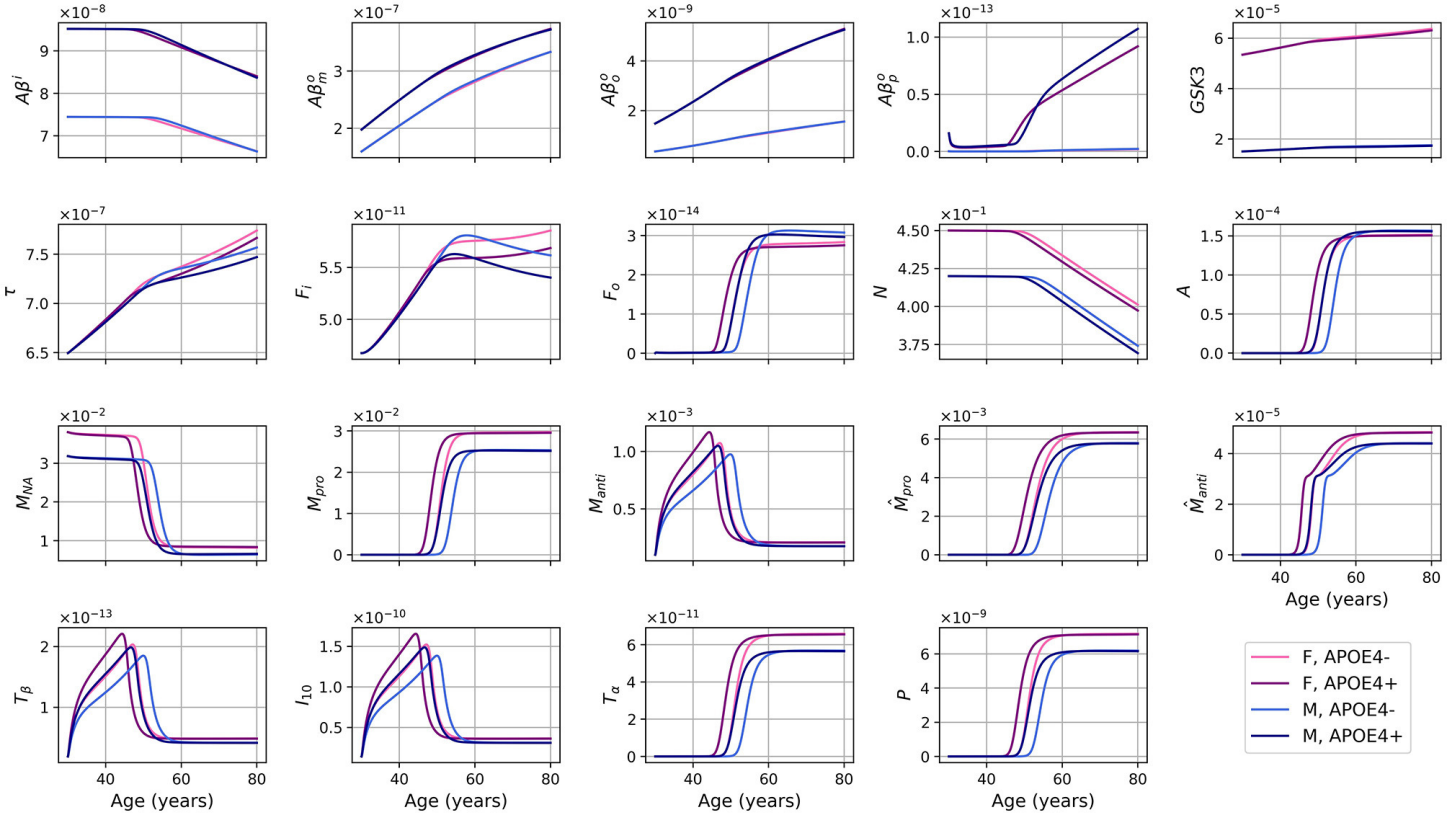
Concentration of each variable, in g/ml or g/cm^3^, as a function of age for every combinations of sex and APOE4 status for our standard model.

The evolution of every variable of our ODE model over 50 years is shown in Figure 2.

For the different forms of Aβ, the curves for APOE4-negative men and women mostly overlap. This is also observed for APOE4-positive individuals. However, it is important to note that despite the overlap, there are noticeable variations in the curves between different groups. Sex appears to have a more significant impact than GSK-3. We also observe a transition to a proinflammatory state occurring around 50 years of age. Women who are APOE4-positive experience this transition earlier, followed by APOE4+ men, APOE4− women, and finally APOE4− men. This transition thus seems mainly influenced by age, then by APOE status, and to a lesser extent by sex.

We observe from Figure 2 that neural loss occurs earlier in individuals with an APOE4 allele regardless of sex. A similar trend is also observed with respect to neural loss between 30 and 80 years old. We obtain losses of 10.84 and 11.70%, for APOE4− and APOE+ women respectively, and of 10.91% and 12.06% for APOE4− and APOE4+ men respectively.

**Figure 3:**
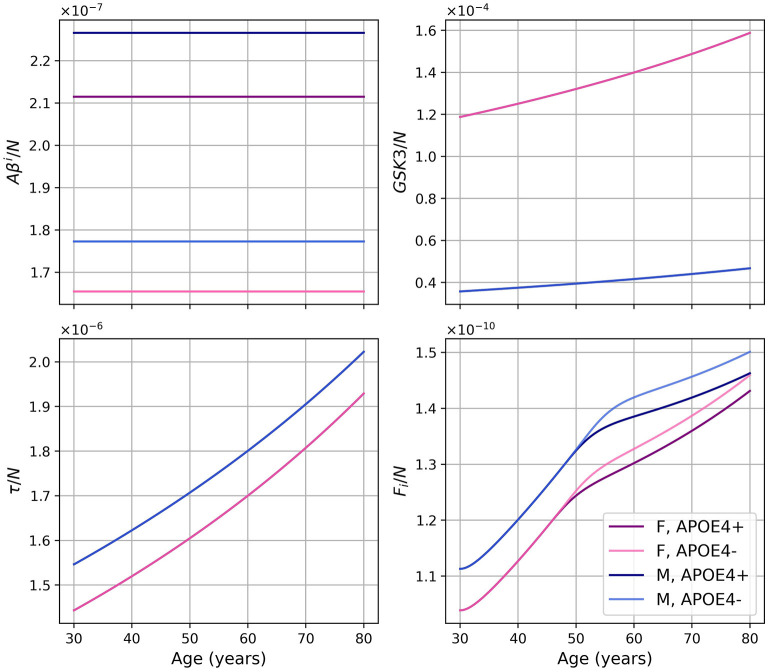
Concentrations of intracellular Aβ (*Aβ*^*i*^), GSK-3 (*G*), phosphorylated/hyperphosphorylated tau proteins (τ), and intracellular NFTs (*F*_*i*_) per unit of neuron density for our standard model.

Dividing the concentrations of intracellular Aβ (*Aβ*^*i*^), GSK-3 (*G*), phosphorylated/hyperphosphorylated tau proteins (τ), and intracellular NFTs (*F*_*i*_) by the density of neurons, we obtain the concentrations per unit density of neurons (Figure 3). We observe that there is almost no variation in the proportion of *Aβ*^*i*^ per unit density of neurons. We also observe that GSK-3 and τ increase almost linearly, which was not the case when we did not divide by the density of neurons.

**Figure 4:**
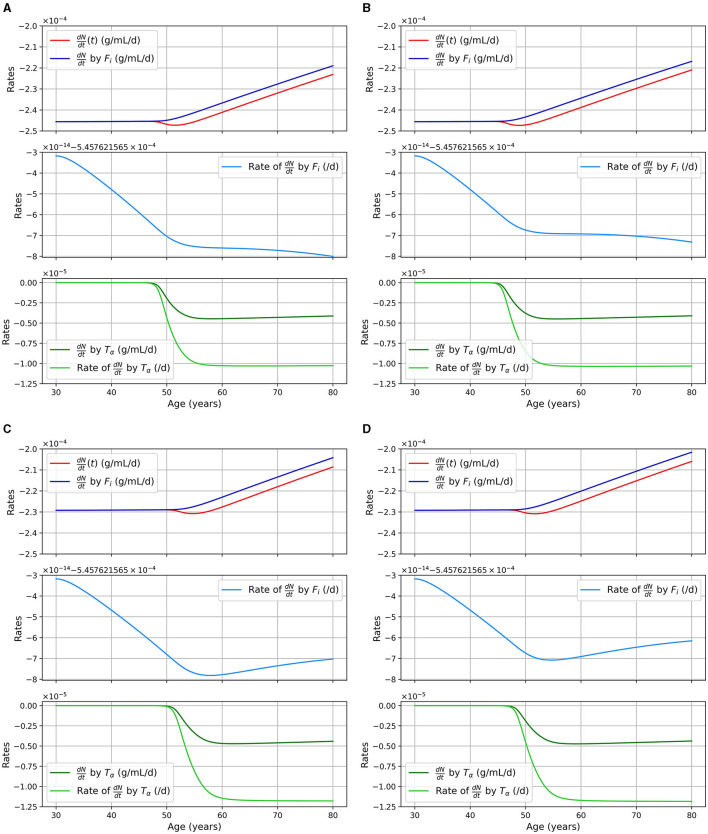
Rate of neuronal death in 1/day or g/cm^3^/day (= g/ml/day), as a function of age, for different combinations of sex and APOE4 status with the standard model. **(A)** Women, APOE4−; **(B)** Women, APOE4+; **(C)** Men, APOE4−; **(D)** Men, APOE4+.


We considered two pathways for neural death, death due to intracellular NFTs and due to proinflammatory cytokines (TNF-α). The second pathway is mitigated by the presence of anti-inflammatory cytokines (IL-10). In Figure 4, we show the rate of neuronal death due to each of these pathways as well as the total rate for different models. The different curves correspond to different portion of the equation for the neural loss, and is as follows:

$$\frac{dN}{dt}=\underset{\text{red}}{\underset{︸}{\underset{\text{dark blue}}{\underset{︸}{\underset{\text{pale blue}}{\underset{︸}{-d_{F_{i}N}\frac{1}{1+\exp(-n\cdot \frac{F_{i}-K_{F_{i}}}{K_{F_{i}}})}}}N}}\underset{\text{dark green}}{\underset{︸}{\underset{\text{pale green}}{\underset{︸}{-d_{T_{\alpha }N}\frac{T_{\alpha }}{T_{\alpha }+K_{T_{\alpha }}}\frac{1}{1+I_{10}/K_{I_{10}}}}}N}}}}.$$

We can observe that neuronal death caused by intracellular NFTs is mainly a function of the density of neural cells. Indeed the pale blue curve of the second subgraph has a variation of the order of 10^−14^/day, it is thus almost constant. We also observe that the total neuronal death (red curves) varies in response to the increase of TNF-α in the model.

**Figure 5:**
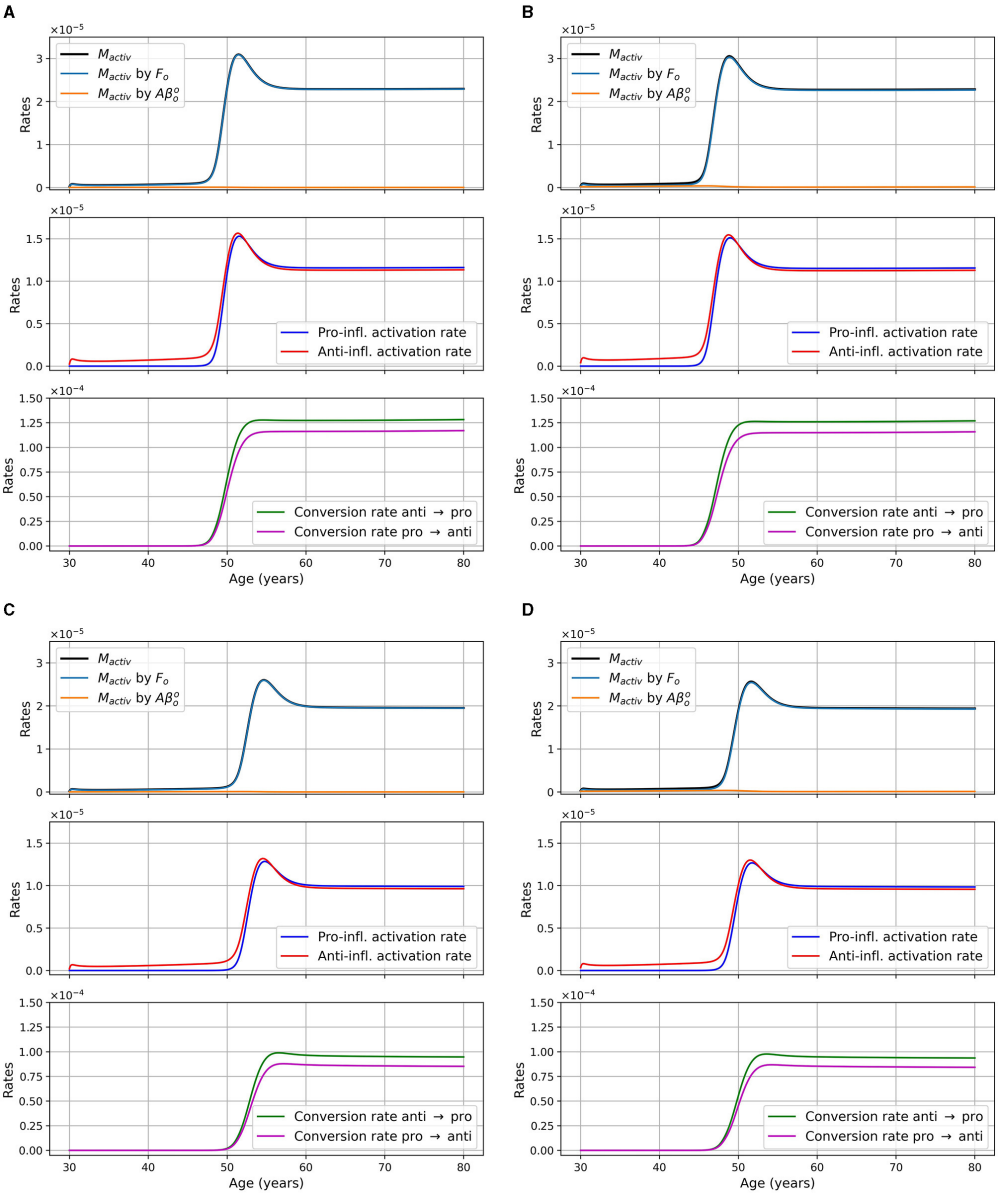
Activation rates of microglia, in g/cm^3^/day, as a function of age for different combinations of sex and APOE4 status. **(A)** Women, APOE4−; **(B)** Women, APOE4+; **(C)** Men, APOE4−; **(D)** Men, APOE4+. Observe that the *M*_*activ*_ curve is very close to the one “*M*_activ_ by *F*_*o*_”.

We monitored microglia activation and its dependence upon different factors. In Figure 5, we display the activation rate of microglia through different pathways. Activation rates are greater and activation occurs earlier in women than in men. Activation also occurs earlier in the presence of APOE4.

### 3.2 Modified models

#### 3.2.1 Models with a constant insulin concentration

In a model where insulin concentration is constant over time, that is Ins(*t*) = Ins_0_ for all *t*, the equation describing the evolution of GSK-3 becomes Equation (26):


(26) $$\begin{array}{c} \frac{dG}{dt}=\lambda_{\text{Ins}G}\frac{N}{N_{0}}-d_{G}G-\frac{G}{N}|\frac{dN}{dt}|. \end{array}$$

**Figure 6:**
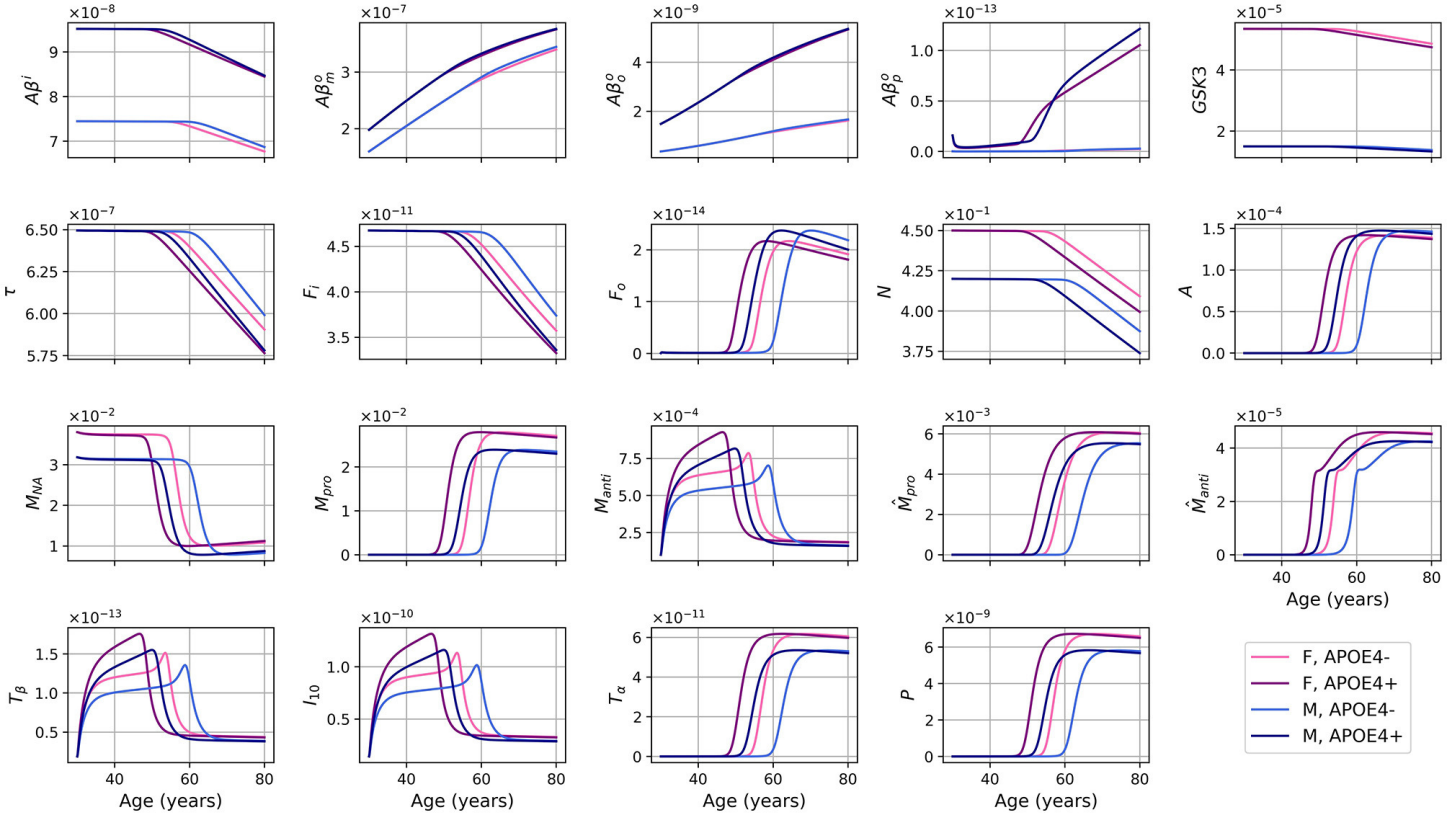
Concentration of each variable, in g/ml or g/cm^3^, as a function of age for every combinations of sex and APOE4 status for the model with constant insulin concentration.

**Figure 7:**
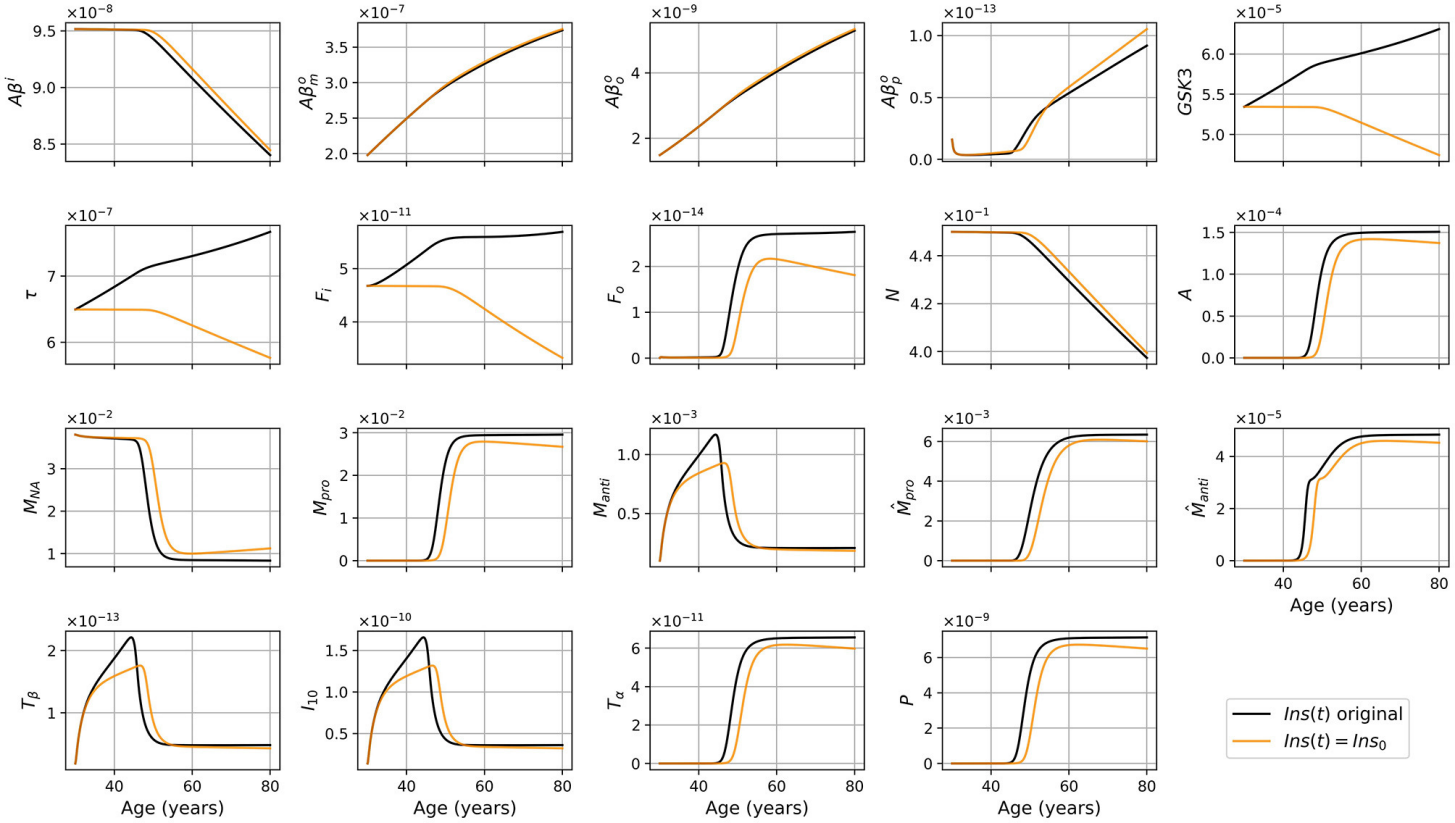
Concentration of each variable, in g/ml or g/cm^3^, as a function of age, for APOE+ women, for the standard model (black), and the model with constant insulin concentration (orange).

The results obtained with this modified version differ significantly from the standard version. For different combinations of sex and APOE4 status, we obtained the results shown in Figure 6. As expected, we no longer observe an increase in GSK-3, tau proteins, and NFTs. The transition to proinflammatory is delayed in the modified model. We also observed a smaller neuronal loss in this modified version. In Figure 7, we compare the predictions of the standard model and of this modified version.

**Figure 8:**
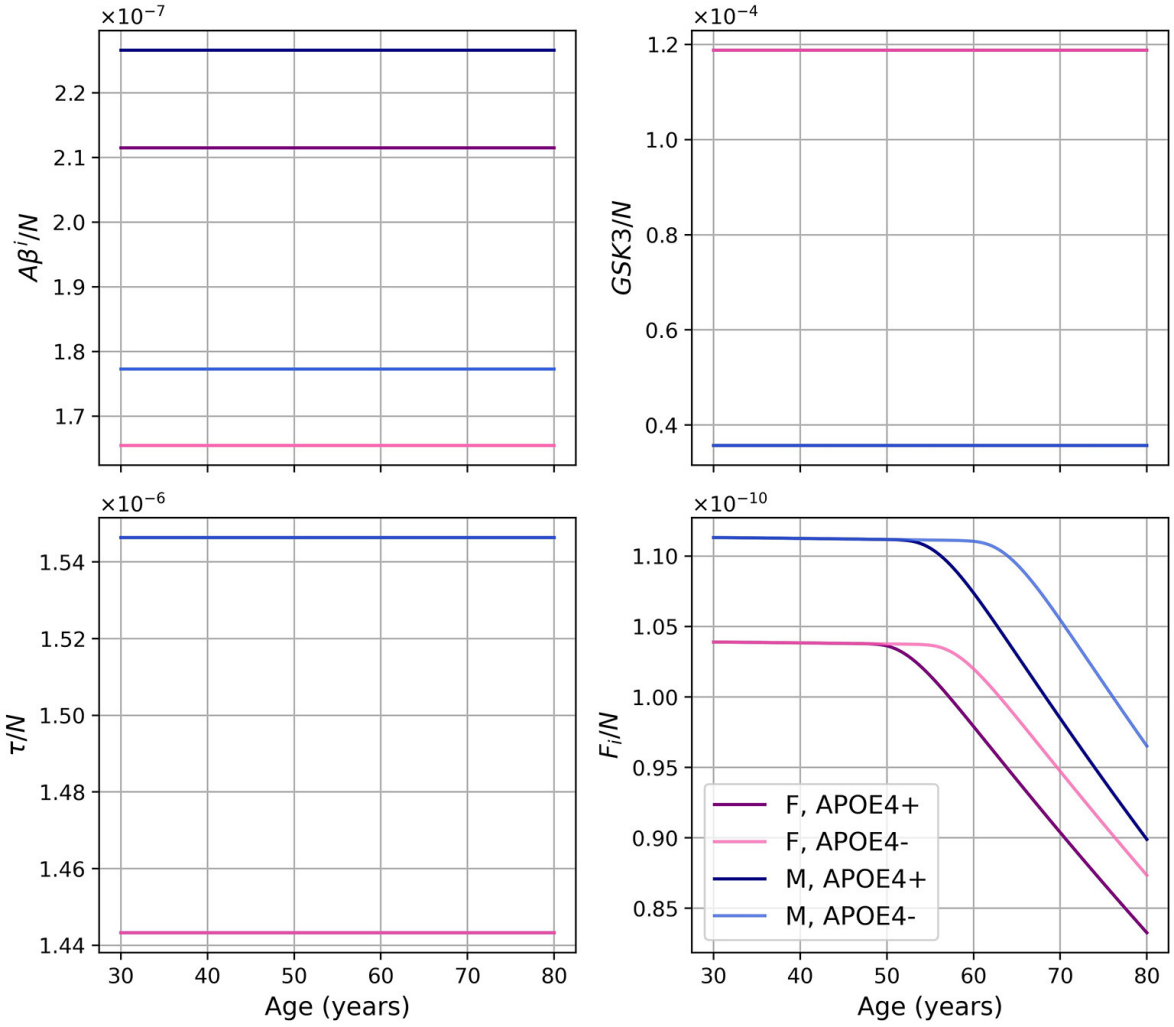
Concentration of intracellular Aβ (*Aβ*^*i*^), GSK-3, phosphorylated/hyperphosphorylated tau proteins (τ), and intracellular NFTs (*F*_*i*_) normalized by the density of neurons, for the model with constant insulin concentration.

As we did for the standard model, we present the concentration of intracellular Aβ (*Aβ*^*i*^), GSK-3, phosphorylated/hyperphosphorylated tau proteins (τ), and intracellular NFTs (*F*_*i*_) normalized by the density of neurons (Figure 8). As was observed in the standard model, there is very little variation for *Aβ*^*i*^/*N*. For this model, this is also the case for *GSK*3/*N* since its creation is due to insulin.

#### 3.2.2 A model with reduced microglia activation rate

**Figure 9:**
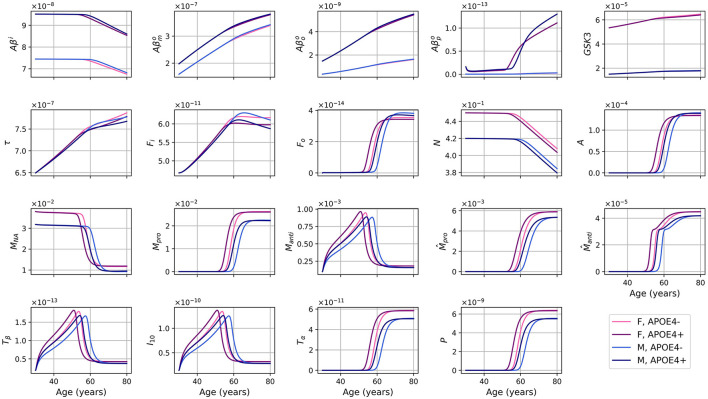
Concentration of each variable, in g/ml or g/cm^3^, as a function of age, for every combinations of sex and APOE4 status, for the model with reduced microglia activation rates (κ_*F*_*o*_*M*_ × 0.5 et $\kappa_{{A\beta }_{o}^{o}M}\times 0.5$).

**Figure 10:**
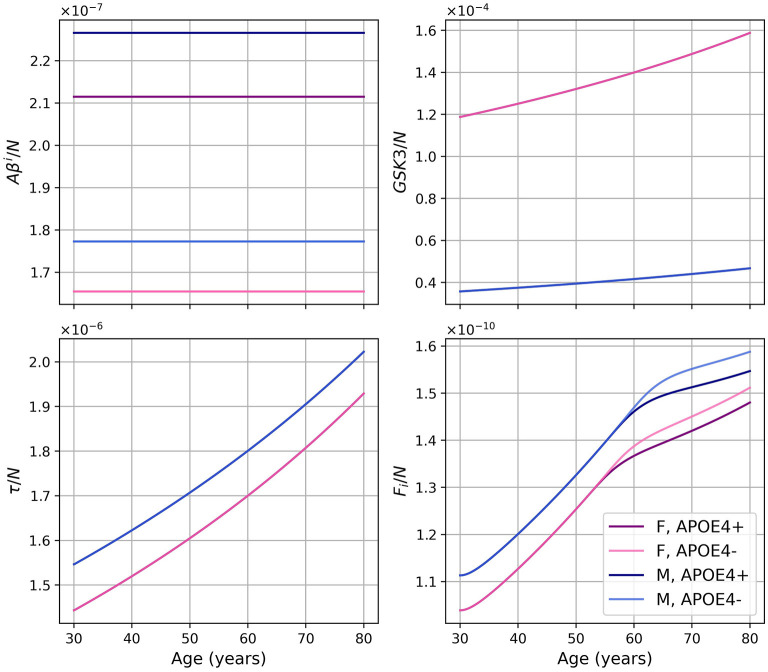
Concentration of intracellular Aβ (*Aβ*^*i*^), GSK-3, phosphorylated/hyperphosphorylated tau proteins (τ), and intracellular NFTs (*F*_*i*_) normalized by the density of neurons for the model with reduced microglia activation rate (κ_*F*_*o*_*M*_ × 0.5 and $\kappa_{{A\beta }_{o}^{o}M}\times 0.5$). The curves for *GSK*3/*N* and τ/*N* for the same sex overlap.

We also made a model where the rate of activation of microglia was reduced by a theoretical intervention with a given efficiency, that is multiplied by ξ where 0 < ξ < 1. With ξ = 0.5 we obtained the results shown in Figures 9, 10.

**Figure 11:**
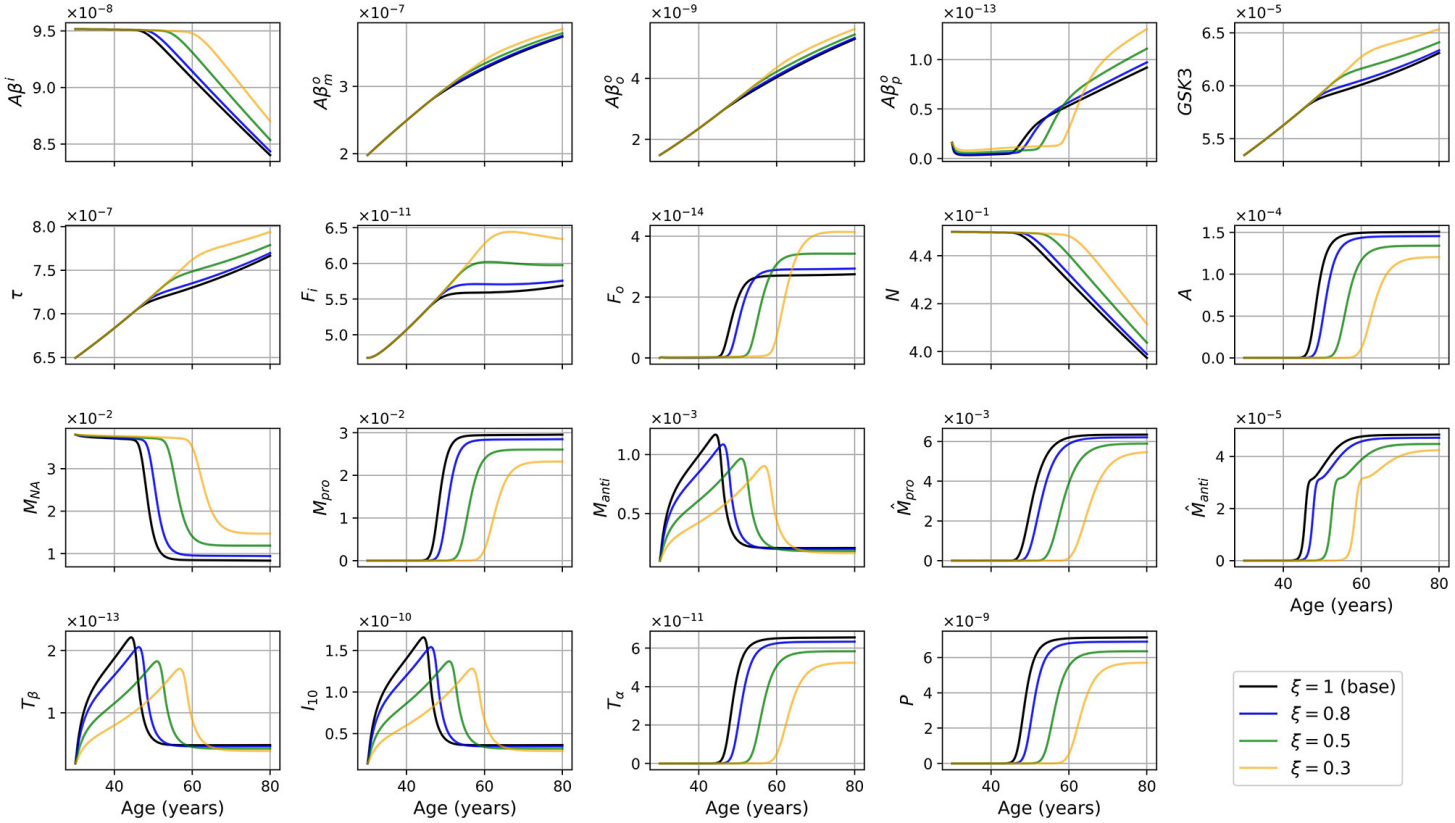
Concentration of each variable, in g/ml or g/cm^3^, as a function of age for APOE+ women, for the standard model (ξ = 1) and for the model with reduced microglia activation rate (ξ = 0.8, 0.5 and 0.3) (κ_*F*_*o*_*M*_ × ξ and $\kappa_{{A\beta }_{o}^{o}M}\times \xi$).

We compared variable concentrations in women with APOE4 between the standard model and the model with reduced microglia activation rate (with ξ = 0.8, 0.5, 0.3) as shown in Figure 11. Results for other combinations of sex and APOE4 status exhibit similar trends. Observe that the smaller the value of ξ, the later the proinflammatory transition occurs (before 50 years old in the standard model and after 60 years old for ξ = 0.3).

**Figure 12:**
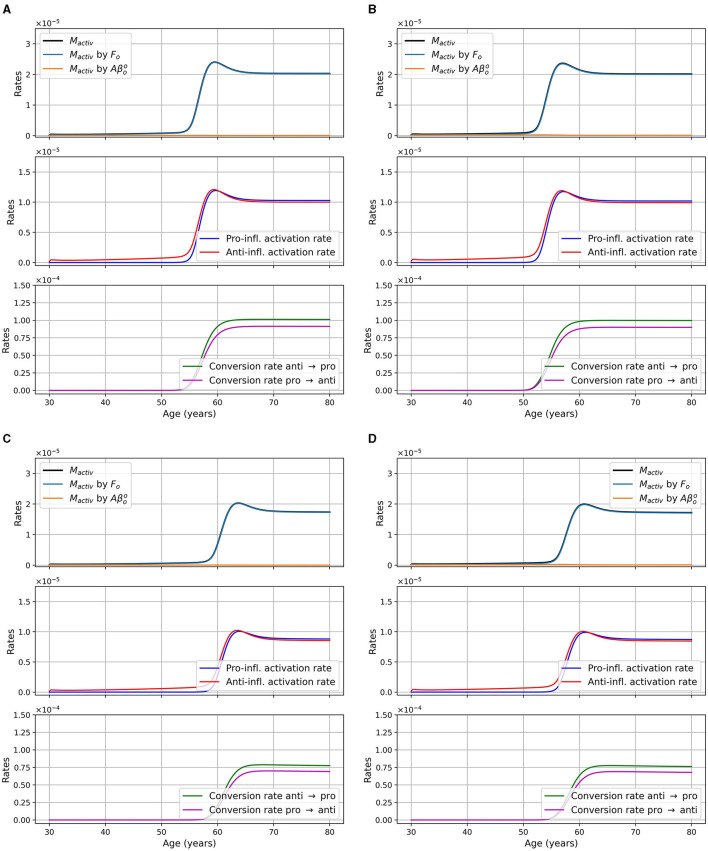
Microglia activation rates in g/cm^3^/day, as a function of age for different combinations of sex and APOE4 status for the model with reduced microglia activation rate: κ_*F*_*o*_*M*_ × 0.5 and $\kappa_{{A\beta }_{o}^{o}M}\times 0.5$ (ξ = 0.5). **(A)** Women APOE4−; **(B)** Women, APOE4+; **(C)** Men, APOE4−; **(D)** Men, APOE4+. The total activation rate *M*_*activ*_ is very similar to the activation rate “*M*_*activ*_ by *F*_*o*_,” so the curves overlap.

We also present the activation rate of microglia through different pathways for the two sex and the two APOE4 statuses for the model with reduced microglia activation rates of κ_*F*_*o*_*M*_ × 0.5 and $\kappa_{{A\beta }_{o}^{o}M}\times 0.5$ in Figure 12. This figure is the analogous of Figure 5 which was for the standard model. We observed that the time at which microglia become activated is delayed when compared to the standard model and that the maxima are smaller.

### 3.3 Implementation

We found that numerical results and running time did not depend significantly on the optional parameters given as an input of the “solve-ivp, BDF” function. While performance was not a criteria for model construction, the model runs within 1 minute on a standard, 3.7 GHz Intel Xeon E5 4-core CPU workstation.

We also determined that our model was robust—i.e., well behaved—to modifying initial conditions in a range of +/- 10% around the central values. Because model sensitivity (i.e., changes in parameters) can identify important elements that are directly related to therapeutical strategies, it is being performed and reported in a second report to respect the page/figure limits of the current submission.

## 4 Discussion

We proposed a mathematical model describing the evolution of multiple markers of brain health through aging, such as amyloid-β, tau proteins, and inflammation. This model behaves differently according to sex and APOE4 status. The general trends of our results (see Figure 2) are consistent with the expected behavior for the different entities of the model, when compared to the literature. First, we observe an anti-inflammatory state that transitions to a proinflammatory state as in Zhang et al. (2021). This transition occurs around 50 years old in our model, and earlier in patients with APOE4. Individuals with APOE4 also accumulated more amyloid plaques, which is consistent with other reports (Liu et al., 2013; Hansen et al., 2018). We also observe that this transition occurs earlier in women, consistent with AD being more prevalent in the latter group (Rajan et al., 2021).

The parameters and initial conditions provided to our standard model were representative of population means for individuals without cognitive impairment. Qualitative validation (see below) shows that when our model evolves through time, most indicators adopt a trajectory leading to inflammation and neural loss, which implies that our population would develop on average a state similar to AD. This is consistent with the interpretation that AD, as primarily an amyloid over-production/under-clearance problem, is a disease process strongly linked to aging, and is present in a large proportion of older individuals. It is important however to underline the gap between pathological expression and cognitive decline; while the two are related, the second is modulated by many other factors such as lifestyle risks, resiliency, and cognitive reserve.

### 4.1 Validation of standard model predictions

#### 4.1.1 Amyloid beta, tau, and NFT

The *qualitative* evolution of variables appears consistent with biological expectations for an aging model (see Figure 2). Contrary to the model of Hao and Friedman (2016), we did not include a specific insult to initiate the AD process. Rather, in the “natural” evolution over 50 years, we observe an increase in the concentrations of different forms of extracellular *Aβ*, tau proteins, and NFTs, demonstrating trends similar to those observed in aging populations. Additionally, these concentrations exhibit patterns that are often associated with AD pathology (Kril et al., 2002; Roher et al., 2009). Our findings suggest that individuals aged 80 years old will, on average, exhibit AD-like pathology, which has been reported extensively; but further research and validation are needed to precisely quantify the levels and their correspondence to real-life cases.

However, when we compare *quantitatively* the model's predicted concentrations of amyloid plaques and phosphorylated/hyperphosphorylated tau proteins with empirical values, we find significant discrepancies. The model predicts much lower concentrations (around 10^−13^ g/ml) for APOE4 negative subjects compared to values reported elsewhere in the literature, of the order of $K_{A\beta_{p}^{o}}=3.11\times 10^{-6}\text{ g/mL}$ (see definition in Supplementary material A, section 1.3). Similarly, the predicted concentration of phosphorylated/hyperphosphorylated tau proteins (10^−7^ g/ml) is notably higher than the observed values in the literature (Schönknecht et al., 2003; Thomann et al., 2009; Duits et al., 2014; Rosén et al., 2014; Magdalinou et al., 2015; Johansson et al., 2017; Gangishetti et al., 2018; Geijselaers et al., 2018; Janelidze et al., 2018; Jeppsson et al., 2019; Nordengen et al., 2019), which are of the order of 10^−11^ g/ml.

The main source of these quantitative discrepancies—and therefore the most likely solution to improve the model's performance—lies in our incorporation of multiple parameters found in animal studies. This became necessary as there were in many instances a dearth of human-specific data. If one were to use the latter, the model's parameters and initial conditions could be better calibrated to match real-life observations, enhancing its ability to accurately simulate AD-like pathology in human populations. This approach would provide a more robust and relevant basis for the model's predictions and enable us to better understand the mechanisms underlying AD progression in humans. Additionally, the use of human data would enable us to consider individual variations, genetic factors, and lifestyle influences that are unique to human populations. By accounting for these factors, we could further refine the model and gain deeper insights into the heterogeneity of AD pathology across different individuals and subpopulations.

#### 4.1.2 Neuronal loss

With our standard model, the percentage of neuronal loss at 80 years old compared to 30 is 10.84 and 11.70%, for women APOE4− and APOE4+, respectively, and 10.91 and 12.06% for men APOE4− and APOE4+, respectively. According to Potvin et al. (2022), the neural loss estimated by volume MRI is 10.70% in women and 12.18% in men. If we assume that this volume loss is mainly due to neural loss, our model yields realistic results (see Table 3).

**Table 3:** Experimental percentages of brain volume loss according to Potvin et al. (2022), and percentages of neurons loss for different versions of our model.

**Sex, APOE4 status**	**Experimental loss of brain volume**	**Standard model**	**Model with Ins(*t*) = Ins(0)**	**Model with ξ = 0.5**
F, APOE4− F, APOE4+	10.70%	10.84%	9.08%	9.34%
		11.70%	11.24%	10.29%
M, APOE4− M, APOE4+	12.18%	10.91%	7.75%	8.45%
		12.06%	10.99%	9.64%

A shortcoming of the current model iteration, however, is related to neuronal death caused by intracellular NFTs. Indeed, we observed in Figure 4 (pale blue curve), the rate of neural death due to intracellular NFT is almost constant with a variation around 10^−14^/day. Thus, with our current parameters, a change in the concentration of *F*_*i*_ has little impact on neural death. As mentioned previously, these parameters would need to be revisited to improve realism.

#### 4.1.3 Activated astrocytes

With respect to the model evolution through time, we observed an increase in the density of activated astrocytes (Morales et al., 2014; Frost and Li, 2017). Furthermore, our model predicted the activation of anti-inflammatory microglia together with anti-inflammatory cytokines (TGF-β and IL-10). This was followed by an increase in the activation of proinflammatory microglia and proinflammatory cytokines (TNF-α and MCP-1), with the deactivation of anti-inflammatory variables. This transition was also observed experimentally *in vivo* (Zhang et al., 2021). We can expect that neurodegeneration is accompanied by an activation of microglia as was seen by many researchers who monitored the level of MHC2, mainly expressed by activated microglia (summarized in Hopperton et al., 2018).

Most of the papers we consulted in the context of this work observed an increase in TGF-β in AD patients when compared to controls. This could however not be investigated here since our models describe brain aging in the absence of a specific insult leading to AD, rather the existence of a state that has many of its hallmarks.

#### 4.1.4 MCP-1, macrophages, and microglia

In section 1.10 of Supplementary material A, the literature we cited stated that MCP-1 concentration in patients with dementia was higher than in controls. It is thus reasonable to observe an increase in of the concentration of this variable in our model with an upward trajectory to a pathological level. We also observe an increase in imported macrophages, both pro- and anti-inflammatory, with a transition toward the proinflammatory state. This occurs with an increase in MCP-1 concentration which recruits macrophages, in agreement with other reports (Deshmane et al., 2009; Das et al., 2015).

Toward the end of the simulation, there are more macrophages and microglia with proinflammatory than anti-inflammatory polarization, and TNF-α is more present than TNF-β and IL-10. This is consistent with the proinflammatory state that we can observe in AD patients (Akiyama et al., 2000).

Quantitatively, the authors in Felsky et al. (2019, figure 2b) estimate that the proportion of activated microglia is between 0.05 and 0.1% in control and AD patients, respe ctively. With our standard model, we obtain that by the end of the simulation, around 80% of the microglia are activated, clearly erroneous.

### 4.2 Validation of constant insulin concentration model predictions

Contrasting the results of the model with constant insulin to the standard model allows us to better understand the impact of insulin.

It is known that insulin concentration varies with age even in cognitively healthy individuals with diabetes (Bryhni et al., 2010). The impact of insulin decrease on GSK-3 is also activity-dependent. Indeed, a decrease in insulin activity implies a decrease in GSK-3 deactivation through the PI3K/Akt/GSK-3β pathway. The activation of GSK-3 then implies an increase of tau proteins hyperphosphorylation (Yang L. et al., 2018; El Sayed et al., 2021).

Also related to insulin, type 2 diabetes (T2D) is a well-known risk factor of AD (Blázquez et al., 2014; Krishnankutty et al., 2017; Yang L. et al., 2018; Zhang et al., 2018). T2D does not cause a decrease in insulin concentration but rather a decrease of its efficiency following insulin resistance (El Sayed et al., 2021). This is accompanied by greater insulin production by beta-pancreatic cells (Abdul-Ghani and DeFronzo, 2021). The two elements form a feedback loop: at some point, insulin production is no longer sufficient to compensate for the resistance (Patel et al., 2004), which results in hyperglycemia, according to which a diagnosis of T2D can be made (Czech, 2017; Abdul-Ghani and DeFronzo, 2021).

The diminution in the efficiency of insulin can be modeled as a diminution of the concentration of efficient insulin. Thus, in our model, the function describing insulin concentration Ins(*t*) corresponds to efficient insulin in non-T2D individuals. Thus, removing the decrease of effective insulin from the model effectively generates a T2D-free model. We would expect that this would lead to a decrease in inflammation and a delay in the onset of the disease since age and T2D are important risk factors for AD. This corresponds indeed to what we observe when comparing the results of our standard model and modified model (Figure 7). We observe that without insulin variation, we obtain less GSK-3, which implies smaller hyperphosphorylation of tau proteins which in turn leads to less intracellular NFT. This can explain why neural death is delayed and why we have a lesser concentration of inflammation-related variables such as microglia, macrophages, and cytokines. We also observe that there is no variation in the graph of *Aβ*^*i*^/*N* (Figure 8) as it was the case with the standard model. This is not surprising as removing changes in insulin concentration has little impact on the variation of *Aβ*^*i*^ and *N* (Figure 7). Thus, results from this insulin experiment are consistent with the fact that increased insulin resistance throughout aging is a risk factor for developing pathology associated with AD.

### 4.3 Validation of reduced microglia activation model predictions

We wished to investigate the efficiency of an eventual therapeutic strategy that would reduce microglia activation rate. We posed ξ ∈ (0, 1] and used κ_*F*_*o*_*M*_ × ξ and $\kappa_{{A\beta }_{o}^{o}M}\times \xi$. We observed that when ξ is closer to 0, the transition to the proinflammatory state and the beginning of neural loss were delayed (see Figure 11). We also observed a smaller activation of anti- and proinflammatory microglia as expected.

Surprisingly, many other variables became less activated in this version of the model including astrocytes (*A*), anti- and proinflammatory macrophages (*M*_*anti*_ and *M*_*pro*_), as well as cytokines and chemokines: TGF-β (*T*_β_), IL-10 (*I*_10_), TNF-α (*T*_α_), and MCP-1 (*P*). This is not totally unexpected since, in our model, inflammation is triggered by the activation of microglia, mainly proinflammatory, which activate the production of TNF-α. This, in turn, is the starting point of a cascade of proinflammatory reactions leading to neuronal death.

In this model version, the concentration of intracellular Aβ remained elevated longer, and the concentrations of extracellular Aβ monomers, oligomers, and plaques, of GSK-3, of phosphorylated/hyperphosphorylated tau proteins, and of intra and extracellular NFTs reached higher maximum values when ξ was smaller (see Figure 11).

Regarding intracellular variables (*Aβ*^*i*^, GSK-3, τ, and *F*_*i*_), we can better understand the time course of these variables by looking at the density of neurons. Indeed, as microglia are less activated, the transition to the proinflammatory state is delayed, which, in turn, delays neural death. In our model, since neural death is the main cause of the decrease in *Aβ*^*i*^, GSK-3, τ, and *F*_*i*_, the time at which these variables started to decrease was delayed. These variables in turn reached greater maxima (Figure 11). When looking at the concentrations normalized by neuron density, we obtained results similar to what we obtained in the standard model with the exception of *F*_*i*_/*N* which has a similar curve but at higher values (Figures 3 vs. 10).

Lastly, we mentioned that the smaller the value of ξ, the more the values of $A\beta_{p}^{o}$ and *F*_*o*_ increase with neuron loss, and the larger is the maximum reached by these variables. The production of these variables is proportional to oligomer concentration which is greater as ξ is smaller. This however does not explain the large variations as a function of ξ. These differences are due to a secondary cause, which is a lesser decrease. Indeed, plaques are degraded by anti-inflammatory microglia and macrophages. As the density of these two cells is smaller for smaller values of ξ, it is expected that this will lead to greater plaque concentration. For extracellular NFTs, we previously mentioned a greater concentration of intracellular NFTs when ξ decreases (compare Figures 3 and 10). Thus, when neurons die, this causes an increase in the concentration of extracellular NFTs. It is not however the only reason explaining the increase in concentration; degradation is also at play here. Indeed, extracellular NFTs are degraded by anti-inflammatory macrophages. As their density reaches a lesser maximum when ξ is smaller there are fewer extracellular NFTs degradation which allows the concentration to reach a greater maximum.

### 4.4 Model limitations

As discussed, the general trend of the curves for our model is consistent with biological expectations. However, quantitative model predictions were not always in line with experimental data. The main issue is related to parameter choice, for which we either could not find good estimations in the scientific literature, or for which values in the literature were not consistent with the rest of the model.

First, parameters related to the activation of microglia κ_*F*_*o*_*M*_ and $\kappa_{{A\beta }_{o}^{o}M}$ were estimated without references (see Supplementary material A, section 1.9). Indeed, we assumed an activation rate of 20% per day based on the hypothesis of Hao and Friedman (2016), according to which the activation is due to a greater extent to extracellular NFT than on Aβ oligomers. We assumed that 2/3 of the activation was due to NFTs and 1/3 to Aβ oligomers. However, there is no guarantee that this is accurate as our simulations yield predictions that differ greatly from measurements reported in Felsky et al. (2019).

Activation of microglia by Aβ oligomers was also very weak compared to its activation by extracellular NFTs. This is due to the fact that the oligomer concentration is of the order of 10^−9^ g/ml and $K_{{A\beta }_{o}^{o}}$ is of the order of 10^−5^ g/ml. Consequently, either the values predicted by the model for the oligomer concentrations are too small, or the value of $K_{{A\beta }_{o}^{o}}$ is too large. In either case, this is not consistent with the observation that oligomers play a role in the activation of microglia (Michelucci et al., 2009; Tang and Le, 2016).

As mentioned in Section 4.1, the value for $K_{{A\beta }_{p}^{o}}$ is too different from the amyloid plaques concentration observed in our model and, as a consequence, the plaques do not cause astrocyte activation. Even if we observe a delay in the onset of inflammation when considering insulin concentration as constant (see Section 4.2), it is clear that the relation between diabetes and insulin concentration could be improved.

The relation between neural death and intracellular NFTs could also be revisited. While in the present model, we consider NFT concentration as equal within each neuron, it would be more realistic to consider that some neurons have a significant quantity of NFTs while others have not (see work by Morsch et al., 1999; Sjögren et al., 2001; Braak and Del Tredici, 2010; Moloney et al., 2021).

The aggregation relationships of amyloid-β could also be revisited. Right now, we consider that it takes at least two monomers to form an oligomer, and at least two oligomers to form a plaque. This is an approximation, because, according to this hypothesis, a plaque (or a fibril, which we have included in the group of plaques) would be composed of at least four monomers (tetramers), but this is much too small, since a tetramer is considered an oligomer. We should therefore find a way to better model the aggregation of Aβ, without necessarily modeling each reaction. Among others, see on this subject Garai and Frieden (2013), Garai et al. (2014), Barz et al. (2018), Man et al. (2019), and Lindstrom et al. (2021). A similar observation can be made with respect to the aggregation of tau proteins. In our model, we consider that tau proteins go directly from monomers to NFT without taking intermediary states into account; this may be an oversimplifying assumption. Discussions on this topic can be found in Rankin et al. (2007), Townsend et al. (2020), and Moloney et al. (2021).

In the section discussing neural density in the Supplementary material A (section 1.7), we defined and discussed the parameter *K*_*I*_10__, while referring to many papers measuring the IL-10 concentration in the CSF (see Table 4 of Supplementary material A). The data point to a greater IL-10 concentration in AD patients than in controls. However, the sources cited in Supplementary Table 4 almost all mention observing no significant differences between concentrations in AD patients and in controls (Tarkowski et al., 2001; Llano et al., 2012; Hu et al., 2019; Rauchmann et al., 2020). Only Stoeck et al. (2005) mentioned a relative increase in AD patients even if other sources observe its increase (e.g., Guillot-Sestier et al., 2015). Thus as from TGF-β, we might have to modify the model to obtain a more realistic description of IL-10 evolution.

Many of our reactions are based on the Michaelis–Menten relation for the rate of an irreversible reaction catalyzed by an enzyme. This has the *K*_*X*_ parameter, corresponding to the substrate concentration for which the reaction rate is half maximum, that is difficult to estimate. In some instances, we used values for parameters *K*_*X*_ that were not based on biological considerations (for example, *K*_*F*_*i*__, $K_{{A\beta }_{o}^{o}}$, and *K*_*P*_). Moreover, the use of the Michaelis–Menten equation is purely empirical and may not be the best for some of our reactions.

## 5 Conclusion

In summary, our study presents a mathematical model that captures the dynamic interplay of multiple variables associated with Alzheimer's disease in control patients aged 30–80 years. This model, based on a system of ordinary differential equations, describes the evolution of multiple variables playing a relevant role in brain aging. It quantitatively represents the evolution of brain health by accounting for complex interactions among multiple biological factors, including amyloid beta, tau proteins, neurons, activated astrocytes, microglia, macrophages, and cytokines. By incorporating relevant literature-derived parameters, we aimed to establish values that reflect a healthy population while considering variations in sex and APOE4 status.

Overall, most variables in our model exhibit trajectories consistent with biological expectations, lending support to its validity. Furthermore, we introduced two modified versions of the model to address specific considerations: one disregarding the impact of type 2 diabetes by assuming a constant insulin concentration, and another reducing the activation rate of microglia. Encouragingly, both modified models yielded results in line with biological plausibility.

However, it is crucial to note that our model's predictions have yet to be validated against experimental data, which represents an essential future step. Additionally, there is room for refinement, particularly in revisiting certain relationships within the model. Once these aspects are addressed, our model holds promise for advancing our comprehension of brain aging in general and AD in particular, facilitating the development of novel therapeutic approaches, and improving the prognostication of disease progression in individual patients.

## Data availability statement

The original contributions presented in the study are included in the article/Supplementary material, further inquiries can be directed to the corresponding author.s

## Author contributions

ÉC: Conceptualization, Investigation, Methodology, Software, Writing—review & editing, Visualization, Writing—original draft. SM: Conceptualization, Investigation, Methodology, Writing—review & editing. ND: Conceptualization, Investigation, Methodology, Supervision, Validation, Writing—review & editing, Formal analysis, Funding acquisition, Project administration, Software. SD: Conceptualization, Funding acquisition, Investigation, Methodology, Project administration, Supervision, Validation, Writing—review & editing, Formal analysis.
