# Supplementary material for: A computational model of Alzheimer's disease at the nano, micro, and macroscales
Source: Front Neuroinform. 2024 Mar 22;18:1348113. doi: 10.3389/fninf.2024.1348113 (PMC10995318; doi:10.3389/fninf.2024.1348113)
Supplement: Supplementary file 1 [file Data_Sheet_1.pdf]

Supplementary Material A

1 PARAMETER ESTIMATION

1.1 Equations for amyloid beta monomer

In equation 5, δ_{APi} quantifies the impact of the presence of the APOE4 gene on the creation of intracellular Aβ. The Aβ42 concentration in brain tissue varies for individuals without APOE4, ranging from 783 ng/g in normal subjects to 5631 ng/g in Alzheimer's disease (AD) patients. Conversely, for individuals with APOE4, this concentration ranges from 2178 ng/g in normal individuals to 8373 ng/g in AD patients (Roher et al., 2009).

To model the temporal increase in Aβ42 concentration, we assume a linear relationship. The corresponding equation is expressed as follows:

$$\delta_{APi} = \frac{\text{Increase with APOE4}}{\text{Increase without APOE4}} - 1 \approx 0.2778. \tag{27}$$

The production rate of Aβ42 is estimated as 3.63×10^{-12} M/s by Lindstrom et al. (2021) in their amyloid aggregation model. According to Raskatov (2019), the molar mass of Aβ42 is $m_{A\beta_m} = 4514\,\text{g/mol}$. Using these values, we can determine the rate constant $\lambda_{A\beta^i}$, which is approximately 1.4157×10^{-6} g/mL/d.

For simplicity, we assume an equal distribution of amyloid produced by neurons, with half being intracellular and half being extracellular. Consequently, we use $\lambda_{A\beta^i} = 7.0787 \times 10^{-7}$ g/mL/d.

The half-life of Aβ in the mouse model is reported to be between 1.5 and 2 hours (Saido and Leissring, 2012; Cirrito et al., 2003; Savage et al., 1998), and we assume that it is similar in humans. Hence, we have $d_{A\beta^i} = \frac{\ln 2}{1{,}75/24\,\text{d}} = 9.5060/\text{d}$.

In equation 6, relying on data from Roher et al. (2009), we determine that $\delta_{APm} = \delta_{APi} = 0.2778$. Additionally, using the information provided by Lindstrom et al. (2021) and assuming that half of the production is intracellular and half is extracellular, we estimate $\lambda_{A\beta^o_m} = \lambda_{A\beta^i} = 7.0787 \times 10^{-7}$ g/mL/d.

Referring to Laird et al. (2005, Figures 2B and 2C), we observe that the production intensity of Aβ42 by neurons is 13 times greater than that of astrocytes, while astrocytes have a production intensity of 0.25 due to lower β-secretase 1 activity. However, activated astrocytes exhibit a higher β-secretase activity, up to 8 times that of non-activated astrocytes (Zhao et al., 2011; Blasko et al., 2000; Frost and Li, 2017). We assume that the production of Aβ in activated astrocytes is 4 times greater than in non-activated astrocytes. Therefore, the production rate of Aβ in activated astrocytes, denoted as $\lambda_{AA\beta^o_m}$, is $\frac{0.25}{13} \times 4 = \frac{1}{13}$ times the production rate in neurons. Consequently, we obtain

$$\lambda_{AA\beta^o_m} = \frac{1}{13}\lambda_{A\beta^o_m} = \frac{1}{13} \times 7.0787 \times 10^{-7}\,\text{g/mL/d} = 5.4451 \times 10^{-8}\,\text{g/mL/d}. \tag{28}$$

The constant $\kappa_{A\beta^o_m A\beta^o_o}$ describes the rate of aggregation of monomers into oligomers and is influenced by the presence of APOE4. If APOE4 is expressed, the rate becomes $\kappa_{A\beta^o_m A\beta^o_o}(1 + AP \cdot \delta_{APmo})$. We assume that the formation of an oligomer requires two monomers, justifying the square power of $A\beta^o_m$.

The article by Garai and Frieden (2013) informs us that the limiting step, between the transition from monomer to dimer or dimer to trimer, is the transition from dimer to trimer. Our equation models the transition from monomer to dimer, but for lack of better data, and for the sake of simplicity, we will take the rate of the rate-limiting step for the value of $\kappa_{A\beta^o_m A\beta^o_o}$. This rate is

provided in units of /(M s) and can be converted to mL/(g d) using the molar mass of Aβ42. As mentioned before, the molar mass of Aβ42 is $m_{A\beta_m} = 4514\,\mathrm{g/mol}$. Thus, we can calculate:

$$\begin{aligned}\kappa_{A\beta^o_m A\beta^o_o} &= 38 \frac{\mathrm{L}}{\mathrm{mol\cdot s}} \left(\frac{1000\,\mathrm{mL}}{1\,\mathrm{L}}\right)\left(\frac{1\,\mathrm{mol}}{2\cdot 4514\,\mathrm{g}}\right)\left(\frac{86\,400\,\mathrm{s}}{1\,\mathrm{d}}\right) \\ &\approx 3.637\times 10^5\,\frac{\mathrm{mL}}{\mathrm{g{\cdot}d}}.\end{aligned} \tag{29}$$

The aggregation of monomers into oligomers occurs 2.7 times faster in subjects with APOE4 compared to those without (Hashimoto et al., 2012). Therefore, we will use $\delta_{APmo} = 2.7 - 1 = 1.7$.

The half-life of $A\beta^o_m$ increases with age, with a value of 3.8 h $\left(= \frac{3.8}{24} = \frac{19}{120}\mathrm{d}\right)$ at 30 years old and 9.4 h $\left(= \frac{9.4}{24} = \frac{47}{120}\mathrm{d}\right)$ at 80 years old (Patterson et al., 2015). We assume that this increase is linear with age. Thus, the half-life as a function of age is given by

$$t_{1/2}(t) = \frac{7}{547500} t + \frac{11}{600}. \tag{30}$$

Finally, the degradation rate is

$$d_{A\beta^o_m}(t) = \frac{\ln 2}{t_{1/2}(t)} = \frac{\ln 2}{\frac{7}{547500} t + \frac{11}{600}}, \quad \text{in day}^{-1}. \tag{31}$$

1.2 Equation for extracellular amyloid beta oligomers

In equation 7, we extend the concept of oligomer aggregation to the formation of plaques, where it is assumed that at least two oligomers are required to form one plaque. This is why the term $A\beta^o_o$ is squared.

To determine the aggregation rate, we refer to Figure 1D in the study by Garai et al. (2014), which illustrates the aggregation of tetramethylrhodamine-labeled Aβ_{1-42} in the presence of different apoE isoforms. Interestingly, the curves corresponding to different forms of the apoE protein, representing distinct APOE alleles, exhibit minimal differences. For rate determination, we use two specific data points from Figure 1D. The initial data point corresponds to the concentration of tetramethylrhodamine-Aβ_{1-42} at 0 h, which is 2 µM. The second data point is obtained after 42 h, where the concentration is observed to be 0.4 times the initial concentration, equivalent to 0.8 µM of tetramethylrhodamine-Aβ_{1-42}. By analyzing these data points, we can estimate the rate of aggregation for further calculations and modeling purposes.

We consider the following differential equation for the aggregation of oligomers into plaques:

$$\frac{dA\beta^o_o}{dt} = -\kappa_{A\beta^o_o A\beta^o_p} (A\beta^o_o)^2. \tag{32}$$

To integrate the equation, we start by separating the variables, resulting in

$$\frac{1}{(A\beta^o_o)^2} dA\beta^o_o = -\kappa_{A\beta^o_o A\beta^o_p} dt.$$

By integrating both sides of the equation, we obtain

$$-\frac{1}{A\beta^o_o(t)} = -\kappa A\beta^o_o A\beta^o_p t + C \quad \Longleftrightarrow \quad \frac{1}{A\beta^o_o(t)} = \kappa A\beta^o_o A\beta^o_p t + K,$$

where C and K are constants. Since at time $t = 0$, we must have:

$$\frac{1}{A\beta^o_o(0)} = K,$$

this implies that the integrated equation is given by:

$$\frac{1}{A\beta_o^o(t)} = \kappa_{A\beta_o^o A\beta_p^o} t + \frac{1}{A\beta_o^o(0)}, \tag{33}$$

where $A\beta_o^o(0)$ represents the initial concentration of $A\beta_o^o$. Isolating $\kappa_{A\beta_o^o A\beta_p^o}$, we obtain

$$\kappa_{A\beta^o{}_o A\beta^o{}_p} = \frac{\frac{1}{A\beta_o^o(t)} - \frac{1}{A\beta_o^o(0)}}{t}. \tag{34}$$

Using $A\beta_o^o(0) = 2\,\mu\mathrm{M}$ and the data point $(42\,\mathrm{h}, 0.8\,\mu\mathrm{M})$, we can now compute the value for $\kappa_{A\beta_o^o A\beta_p^o}$.

$$\kappa_{A\beta_o^o A\beta_p^o} = \frac{\frac{1}{0.8\,\mu\mathrm{M}} - \frac{1}{2\,\mu\mathrm{M}}}{1.75\,\mathrm{d}} = \frac{3}{7}\,\text{per-mode=power}/(\mu\mathrm{M\,d}) \approx 4.2857 \times 10^5/(\mathrm{M\,d}). \tag{35}$$

Considering that an oligomer consists of at least two monomers, its molar mass is at least $2 \times m_{A\beta_m} = 2 \times 4514\,\mathrm{g/mol} = 9028\,\mathrm{g/mol}$ (Raskatov, 2019). Therefore, we can calculate the value of $\kappa_{A\beta_o^o A\beta_p^o}$ as follows:

$$\kappa_{A\beta_o^o A\beta_p^o} = 4.2857 \times 10^5\,\frac{\mathrm{L}}{\mathrm{mol{\cdot}d}}\left(1000\,\frac{\mathrm{mL}}{\mathrm{L}}\right)\left(\frac{1\,\mathrm{mol}}{9028\,\mathrm{g}}\right) = 4.7471 \times 10^4\,\frac{\mathrm{mL}}{\mathrm{g{\cdot}d}}. \tag{36}$$

The constant $d_{A\beta_o^o}$ represents the rate of degradation. According to Garai and Frieden (2013), the rate at which trimers convert to dimers is limiting compared to the rate at which dimers degrade into monomers ($(0.3 \pm 0.1) \times 10^{-3}$ /s versus $(12.7 \pm 3.0) \times 10^{-3}$ /s). Thus, we can calculate $dA\beta_o^o$,

$$d_{A\beta_o^o} = 0.3 \times 10^{-3}/\mathrm{s} \cdot \left(\frac{86\,400\,\mathrm{s}}{1\,\mathrm{d}}\right) = 25.92/\mathrm{d}. \tag{37}$$

1.3 Equation for extracellular amyloid beta plaques

In equation 8, $d_{M_{anti}A\beta_p^o}$ and $d_{\hat{M}_{anti}A\beta_p^o}$ are the minimal rates of degradation of Aβ plaques by microglia and macrophages anti-inflammatory, respectively. We refer to Figure 5 of DeWitt et al. (1998) and Figure 1A of Majumdar et al. (2008) to get the half-life of plaques in presence of microglia and macrophages, respectively. By using their data, we determine that

$$d_{M_{anti}A\beta_p^o} = \frac{\ln 2}{3\,\mathrm{d}} = 0.2310/\mathrm{d}, \tag{38}$$

and

$$d_{\hat{M}_{anti}A\beta_p^o} = \frac{\ln 2}{0.85\,\mathrm{d}} = 0.8155/\mathrm{d}. \tag{39}$$

This is coherent with the fact that macrophages are more efficient at degrading plaques than microglia (Lai and McLaurin, 2012; Thriault et al., 2015).

The degradation of plaques is found to be reduced in individuals with APOE4. According to Zhao et al. (2009), the degradation of Aβ42 after 48 hours of incubation was approximately 50% in PDAPP transgenic mice brain sections with macrophages expressing the APOE2 allele, while it decreased of 20% with APOE3 and nearly 0% with APOE4. Based on the observations from Zhao et al. (2009, Fig. 4A, B), we estimate the degradation rate in individuals with APOE4 to be 5% of the normal rate. Assuming a linear degradation model, we conclude that the degradation rate is four times slower (5%/20%) in subjects with APOE4 compared to APOE3. Thus, we obtain

$$1 + \delta_{APdp} = 0.25 \quad \Longrightarrow \quad \delta_{APdp} = -0.75.$$

We assume a similar relationship for microglia, where the factor $\left(1 + AP \cdot \delta_{APdp}\right)$ multiplies both degradation rates. According to Roberts et al. (2017, Table 1), plaque concentration is determined

using formic acid and urea/detergent (see Roberts et al. (2017, Figure 6)). The volume of gray matter in controls is $(527.4 \pm 26.8)\,\mathrm{cm}^3$, corresponding to an average of 527.4 mL. For controls, we have

$$K_{A\beta_p^o} = \frac{(1,11+0,53)\,\mathrm{mg}}{527.4\,\mathrm{mL}} \left(\frac{1\,\mathrm{g}}{1000\,\mathrm{mg}}\right) = 3.11 \times 10^{-6}\,\mathrm{g/mL}. \tag{40}$$

1.4 Equation for glycogen synthase kinase 3 (GSK-3)

In equation 9, we assume that cerebral insulin is predominantly derived from the periphery (Gray et al., 2014; Wallum et al., 1987). The peripheral insulin curve is based on data from Bryhni et al. (2010). We used estimated the values of the two first graphs of their Figure 1, presenting the median insulin concentration in men and women, respectively, as a function of age, for people not declared diabetic. We used the molar mass of human insulin, that is 5808 Da (Litwack, 2022), which is equivalent to 5808 g/mol, to convert their data from pmol/L to g/mL. By performing a linear regression on the data, we derive the following equations for peripheral insulin concentration (in g/mL):

$$\mathrm{Ins}_{\mathrm{peri}}^F(t) = -4.151 \times 10^{-15} \cdot t + 3.460 \times 10^{-10}, \tag{41}$$

$$\mathrm{Ins}_{\mathrm{peri}}^M(t) = -4.257 \times 10^{-15} \cdot t + 3.763 \times 10^{-10}, \tag{42}$$

where t is given in days. Next, we established the functions of brain insulin concentration as a function of age. The concentration in the brain is approximately 10 times lower than that in the periphery (Gray et al., 2014; Kern et al., 2006; also observed by Wallum et al., 1987), thus we set $\mathrm{Ins}^X(t) = 0.10 \cdot \mathrm{Ins}_{\mathrm{peri}}^X(t)$. Therefore, we have

$$\mathrm{Ins}^F(t) = 0.10 \cdot \mathrm{Ins}_{\mathrm{peri}}^F(t) = -4.151 \times 10^{-16} \cdot t + 3.460 \times 10^{-11}, \tag{43}$$

$$\mathrm{Ins}^M(t) = 0.10 \cdot \mathrm{Ins}_{\mathrm{peri}}^M(t) = -4.257 \times 10^{-16} \cdot t + 3.763 \times 10^{-11}, \tag{44}$$

expressed in g/mL, where t is measured in days.

GSK-3 activation is increased by the decrease in insulin concentration or activity (Jolivalt et al., 2008; Yang et al., 2018; El Sayed et al., 2021). The reduction in insulin activity triggers a greater activation of GSK-3 through the PI3K/Akt/GSK-3β signaling pathway (Yang et al., 2018; El Sayed et al., 2021). Therefore, the rate of GSK-3 activation at age t is determined by the basal rate ($\lambda_{\mathrm{Ins}G}$) multiplied by the initial insulin concentration divided by the concentration at age t. We observe that $\frac{\mathrm{Ins}_0}{\mathrm{Ins}(t)} \geq 1$, indicating that the rate increases as insulin activity declines.

We assumed that the normal insulin concentration, Ins_0, corresponds to the concentration at 30 years (10 950 d). Based on the insulin function definition, we obtain

$$\mathrm{Ins}_0^F = \mathrm{Ins}^F(10950) = -4.151 \times 10^{-16} \cdot 10950 + 3.460 \times 10^{-11} = 3.006 \times 10^{-11}\,\mathrm{g/mL}, \tag{45}$$

$$\mathrm{Ins}_0^M = \mathrm{Ins}^M(10950) = -4.257 \times 10^{-16} \cdot 10950 + 3.763 \times 10^{-11} = 3.296 \times 10^{-11}\,\mathrm{g/mL}, \tag{46}$$

for females and males, respectively.

Regarding degradation, we found that the half-life of GSK-3 is 41 ± 4 hours (Domnguez et al., 2012). Thus, we have

$$d_G = \frac{\ln 2}{41/24} = 0.4057/\mathrm{d}. \tag{47}$$

To determine the rate of GSK-3 creation or activation by insulin, $\lambda_{\mathrm{Ins}G}$, we will consider the equilibrium of the equation. We first need the normal concentration of the kinase, G_0. Based on studies in healthy human controls, the GSK-3 density in brain tissue is (1104 ± 86) fmol/mg for females and (310 ± 42) fmol/mg for males (Knight et al., 2021). The molar mass of GSK-3β is 47 000 g/mol (Pandey and DeGrado, 2016), and the brain density is ρ_{br} =

$1.03\,\mathrm{g/mL}$ (National Institute of Standards and Technology, 2017). This leads to the following concentrations:

$$G_0^F = 1104 \times 10^{-12}\,\frac{\mathrm{mol}}{\mathrm{g_{brain}}}\left(47\,000\,\frac{\mathrm{g}}{\mathrm{mol}}\right)\left(1.03\,\frac{\mathrm{g_{brain}}}{\mathrm{mL}}\right) = 5.3445 \times 10^{-5}\,\mathrm{g/mL}, \tag{48}$$

$$G_0^M = 310 \times 10^{-12}\,\frac{\mathrm{mol}}{\mathrm{g_{brain}}}\left(47\,000\,\frac{\mathrm{g}}{\mathrm{mol}}\right)\left(1.03\,\frac{\mathrm{g_{brain}}}{\mathrm{mL}}\right) = 1.5007 \times 10^{-5}\,\mathrm{g/mL}, \tag{49}$$

for females and males, respectively. We want to have equilibrium of equation (9) at a time t_0, i.e. $\frac{dG}{dt}(t_0) = 0$, if $G(t_0) = G_0$, $\mathrm{Ins}(t_0) = \mathrm{Ins}_0$, $N(t_0) = N_0$, and neuronal death is negligible ($\frac{dN}{dt} \approx 0$).

$$\frac{dG}{dt} = \lambda_{\mathrm{Ins}G}\underbrace{\frac{\mathrm{Ins0}}{\mathrm{Ins}(t_0)}}_{=1}\underbrace{\frac{N(t_0)}{N_0}}_{=1} - d_G\underbrace{G(t_0)}_{=G_0} - \frac{G(t_0)}{N(t_0)}\underbrace{\left|\frac{dN}{dt}\right|}_{\approx 0} = 0. \tag{50}$$

We isolate $\lambda_{\mathrm{Ins}G}$ in order to satisfy the equation, we find

$$\lambda_{\mathrm{Ins}G} = d_G G_0. \tag{51}$$

This allows us to determine the following rates:

$$\lambda_{\mathrm{Ins}G}^F = d_G G_0^F = 0.4057/\mathrm{d} \cdot 5.3445 \times 10^{-5}\,\mathrm{g/mL} = 2.1685 \times 10^{-5}\,\mathrm{g/mL/d}, \tag{52}$$

$$\lambda_{\mathrm{Ins}G}^M = d_G G_0^M = 0.4057/\mathrm{d} \cdot 1.5007 \times 10^{-5}\,\mathrm{g/mL} = 6.0891 \times 10^{-6}\,\mathrm{g/mL/d}, \tag{53}$$

for females and males, respectively.

1.5 Equation for phosphorylated/hyperphosphorylated tau proteins

In equation 10, λ_τ represents the initial rate of this hyperphosphorylation. Sato et al. (2018) obtained (26.3 ± 9.2) pg/mL/d, hence we adopt $\lambda_\tau = 26.3 \times 10^{-12}\,\mathrm{g/mL/d}$.

The ratio of microtubule-bound tau (τ_A) to soluble (phosphorylated) tau (τ_P) in neurons undergoes a change from 4.7 to 1.1 for control neurons and those infected with GSK-3β S9A, respectively, as observed in Hong and Lee (1997, figure 2D). The experiment duration for these results is assumed to be 12 h (0.5 d). Additionally, Iqbal et al. (2010a) reports an approximate intraneuronal tau concentration of 2 µM. So,

$$\tau_A + \tau_P = 2\,\mu\mathrm{M} \quad\Longleftrightarrow\quad \tau_A = 2\,\mu\mathrm{M} - \tau_P. \tag{54}$$

We then have

$$\begin{aligned} \frac{\tau_A^N}{\tau_P^N} = 4.7 \quad &\Longleftrightarrow \quad \frac{2\,\mu\mathrm{M} - \tau_P^N}{\tau_P^N} = 4.7 \\ &\Longleftrightarrow \quad \tau_P^N = \frac{20}{57} \approx 0.35\,\mu\mathrm{M}, \end{aligned} \tag{55}$$

for normal neurons. Similarly, for infected neurons, we have $\tau_P^I = \frac{20}{21} \approx 0.95\,\mu\mathrm{M}$. Assuming an approximation by temporarily neglecting other terms in the equation, we obtain $\frac{d\tau}{dt} = \lambda_{G\tau}\frac{G}{G_0}$. Notably, in Hong and Lee (1997), figures 2A and 2B reveal significant differences in GSK-3 stainings between GSK3 S9A cells and LacZ cells. Let's consider infected neurons with a GSK-3 concentration 1000 times higher than that of the controls.

Thus, on the one hand, we have

$$\frac{d\tau}{dt} = \frac{\tau_P^I - \tau_P^N}{dt} = \frac{0.95\,\mu\text{M} - 0.35\,\mu\text{M}}{0.5\,\text{d}} = 1.2\,\mu\text{M/d}, \tag{56}$$

and on the other hand,

$$\lambda_{G\tau}\frac{G}{G_0} = \lambda_{G\tau}\cdot 1000. \tag{57}$$

Hence, we obtain the following approximate rate:

$$\lambda_{G\tau} = \frac{1.2\,\mu\text{M/d}}{1000} = 0.0012\,\mu\text{M/d} = 1.2\times 10^{-9}\,\frac{\text{mol}}{\text{L}\cdot\text{d}}. \tag{58}$$

The molar mass of phosphorylated tau is between 65 and 80 kDa (Sjgren et al., 2001); taking 72.5 kDa, i.e., 72 500 g/mol, we obtain the rate as follows:

$$\lambda_{G\tau} = 1.2\times 10^{-9}\,\frac{\text{mol}}{\text{L}\cdot\text{d}}\left(\frac{1\,\text{L}}{1000\,\text{mL}}\right)\left(\frac{72\,500\,\text{g}}{1\,\text{mol}}\right) \approx 8.72\times 10^{-8}\,\text{g/mL/d}. \tag{59}$$

For the value of the normal kinase concentration, G_0, we use the values obtained in the previous section for GSK-3 (Section 1.4, equations (48) and (49)). We have

$$G_0^F = 5.3445\times 10^{-5}\,\text{g/mL},$$
$$G_0^M = 1.5007\times 10^{-5}\,\text{g/mL},$$

for females and males, respectively.

Hyperphosphorylated tau proteins have a tendency to aggregate and form intracellular neurofibrillary tangles (NFTs), denoted as F_i. To capture this process in equation (10), we included the term $-\kappa_{\tau F_i}(\tau)^2\frac{N}{N_0}$. This term accounts for the reaction where the aggregation of NFTs is influenced by the squared concentration of hyperphosphorylated tau proteins (τ), indicating that at least two tau proteins are necessary for one NFT formation. According to Townsend et al. (2020), tau aggregation rates were studied in the presence of heparin, an analog of heparan sulfate. It was observed that tau filaments colocalize with heparan sulfate proteoglycans *in vivo* (Townsend et al., 2020; Alavi Naini and Soussi-Yanicostas, 2018). For heparin concentrations of 2.0 µM, 5.0 µM, and 10.0 µM, the primary nucleation rates were 32×10^{-6}/(M s), 35×10^{-6}/(M s), and 33×10^{-6}/(M s), respectively (Townsend et al., 2020, Table 1). The average rate obtained from these values is $33.\overline{3}\times 10^{-6}$/(M s). The study used the Δtau187 form of tau, which is a truncated version consisting of residues 255 to 441 from the N2R4 tau isoform. The molar mass of tau isoforms shows a linear relationship with the number of amino acids (Goedert and Jakes, 1990). For instance, tau441, the longest isoform, has a molar mass of approximately 45 850 g/mol, equivalent to about 103.9683 g/mol per amino acid. Similarly, tau352 has a molar mass of approximately 36 760 g/mol, or 104.4318 g/mol per amino acid. Assuming a molar mass of 104 g/mol per amino acid, we estimate the molar mass of Δtau187 to be approximately 19 344 g/mol (104 g/mol · (441 − 255)).

Therefore,

$$\kappa_{\tau F_i} = 33.\overline{3}\times 10^{-6}\text{per-mode=fraction, inter-unit-product=}\cdot\text{L/(mol s)}\left(\frac{1\,\text{mol}}{19\,344\,\text{g}}\right)\left(\frac{86\,400\,\text{s}}{1\,\text{d}}\right)\left(\frac{1000\,\text{mL}}{1\,\text{L}}\right)\approx \tag{60}$$

The half-life of phosphorylated tau proteins in neurons derived from human induced pluripotent stem cells is (5.16 ± 1.28) days (Sato et al., 2018). Therefore, we take

$$d_\tau = \frac{\ln 2}{5.16\,\text{d}} = 0.1343/\text{d}. \tag{61}$$

1.6 Equations for NFTs

In equation (11), d_{F_i} refers to the rate of degradation and elimination of intracellular NFTs. The intracellular NFTs can not be degraded by the host neuron, they accumulate gradually in the cytoplasm (Braak and Del Tredici, 2010). Hence, we assume a very low rate, following the idea of Hao and Friedman (2016), this yields

$$d_{F_i} = \frac{1}{10^2} \cdot d_\tau = \frac{0.1343}{10^2} = 1.343 \times 10^{-3}/\text{d}. \tag{62}$$

In equation (12), the constant κ_{MF_o} represents the maximum degradation rate of extracellular NFTs by microglia, while $K_{M_{anti}}$ denotes the concentration of anti-inflammatory microglia at which the degradation rate is half-maximal. In the study by Luo et al. (2015), they observed a degradation of 80% of SI-tau, with an initial concentration of $1\,\mu\text{g/mL}$, using 3×10^5 microglia per well in a 48-well plate over a 48 hour period. Based on this, we assume a maximum degradation rate of 40% per day, setting $\kappa_{MF_o} = 0.4/\text{d}$.

We determine the density of microglia using the findings of Pelvig et al. (2008) as provided in Table 2. The calculations yield:

$$M_0^F = \eta_{M^F} \times \rho_{\text{br}} = \frac{3.7}{100} \times 1.03 = 3.811 \times 10^{-2}\,\text{g/cm}^3, \tag{63}$$

$$M_0^M = \eta_{M^M} \times \rho_{\text{br}} = \frac{3.1}{100} \times 1.03 = 3.193 \times 10^{-2}\,\text{g/cm}^3. \tag{64}$$

Here, η_M represents the percentage of brain cells that are microglia, and we use the value from Table 2.

We make the assumption that the degradation rate reaches half of its maximum when half of the population is activated. Additionally, we assume that half of the activated population has an anti-inflammatory polarization. Therefore, we obtain the following expression:

$$K^F_{M_{anti}} = \frac{1}{4} M_0^F = \frac{1}{4} \cdot 3.811 \times 10^{-2}\,\text{g/cm}^3 = 9.5275 \times 10^{-3}\,\text{g/cm}^3, \tag{65}$$

$$K^M_{M_{anti}} = \frac{1}{4} M_0^M = \frac{1}{4} \cdot 3.193 \times 10^{-2}\,\text{g/cm}^3 = 7.9825 \times 10^{-3}\,\text{g/cm}^3. \tag{66}$$

We assume that the rate of degradation of extracellular NFTs is lower than that of hyperphosphorylated tau proteins ($d_{F_o} < d_\tau$), but higher than the degradation rate of intracellular NFTs ($d_{F_o} > d_{F_i} = 10^{-2} \cdot d_\tau$). Based on these assumptions, we will use the following expression:

$$d_{F_o} = \frac{1}{10} \cdot d_\tau = \frac{1}{10} \cdot 0.1343/\text{d} = 1.343 \times 10^{-2}/\text{d}. \tag{67}$$

1.7 Equation for the density of neurons

In Schwab et al. (1999), the author studies the longevity of neurons with NFTs and obtains a mean survival time of 2.51 years which gives

$$d_{F_iN} = \frac{1}{916.15\,\text{d}} = 1.0915 \times 10^{-3}/\text{d}. \tag{68}$$

In Khatoon et al. (1992), the authors measure the mean concentration of hyperphosphorylated tau proteins in the grey matter and obtain $6.0\,\text{ng}/\mu\text{g}$ of brain homogenate. Recalling that the brain density is $\rho_{\text{br}} = 1.03\,\text{g/cm}^3$, we get

$$6.0\,\frac{10^{-9}\,\text{g}_{\text{p-tau}}}{10^{-6}\,\text{g}_{\text{br}}}\left(1.03\,\frac{\text{g}_{\text{br}}}{\text{cm}^3}\right) = 6.18 \times 10^{-3}\,\text{g}_{\text{p-tau}}/\text{cm}^3.$$

A maximum proportion of 40% of tau proteins in the brain of AD patients is non-fibrillated (Kpke et al., 1993; Iqbal et al., 2010b), so approximately 60% is fibrillated. We can thus estimate

the intracellular NFT concentration in AD patients by

$$0.6 \cdot 6.18 \times 10^{-3}\,\mathrm{g/cm^3} = 3.708 \times 10^{-3}\,\mathrm{g/cm^3} = 3.708 \times 10^{-3}\,\mathrm{g/mL}. \tag{69}$$

We assume that the hyperphosphorylated tau concentration for which the rate of neural death caused by intracellular NFTs is half-maximal is ten times smaller than the concentration in AD patients. This is justified by the fact that very little hyperphosphorylated tau has been detected in controls (Khatoon et al., 1992). We thus have

$$K_{F_i} = 0.1 \cdot 3.708 \times 10^{-3}\,\mathrm{g/mL} = 3.708 \times 10^{-4}\,\mathrm{g/mL}. \tag{70}$$

With the model, We obtain an intracellular NFT concentration of about 10^{-11} g/mL. Therefore, taking a value of K_{F_i} in the order of magnitude of 10^{-4} g/mL would result in minimal impact on neural death caused by intracellular NFTs. Instead, we opt for a value of $K_{F_i} = 1.25 \times 10^{-10}$ g/mL, which allows for a more significant effect. To achieve a steep dependency of the neural death rate on F_i, we set the sigmoid coefficient n to 15. This choice introduces a delay in the death of the first neurons and implies that neural loss will not be linear from a young age, as we can observe with the data of Potvin et al. (2022).

According to Potvin et al. (2022), the greatest annual brain volume loss occurs in women aged 90 to 100, with an average rate of 0.726% per year. Assuming this loss corresponds to one-tenth (1/10) of the maximum neural loss caused by TNF-α, we employ this value in our calculations. Hence, we use the value

$$d_{T_\alpha N} = \frac{7,26 \times 10^{-3}}{365\,\mathrm{d}} \cdot 10 \approx 1.989 \times 10^{-4}/\mathrm{d}. \tag{71}$$

The concentration of TNF-α in the cerebrospinal fluid (CSF) in control and in patients with AD varies greatly in literature (for controls: Csuka et al., 1999; Jensen et al., 2019; Liu et al., 2022; for controls and AD patients: Garlind et al., 1999; Hesse et al., 2016; Hu et al., 2019; Jia et al., 2005; Llano et al., 2012; Rauchmann et al., 2020; Richartz et al., 2005; Tarkowski et al., 1999). We mainly find average values ranging from 0.2 to 20.56 pg/mL in controls and between 0.30 to 52.23 pg/mL in patients with AD.

In the study by Tarkowski et al. (1999), the average concentration of TNF-α in AD patients was found to be approximately 300 pg/mL, which is about 25 times higher than the average concentration in controls (13.2 pg/mL). Some sources support the notion of increased TNF-α concentration in AD patients compared to controls (Tarkowski et al., 1999; Jia et al., 2005; Hu et al., 2019). However, other sources suggest either lower concentrations (Richartz et al., 2005) or no significant difference (Garlind et al., 1999; Lanzrein et al., 1998; Hesse et al., 2016). Lanzrein et al. (1998) reported a decrease in TNF-α levels in certain brain regions (frontal cortex, superior temporal gyrus, entorhinal cortex) of AD patients compared to controls. In contrast, Zhao et al. (2003) reported higher levels of TNF-α in AD patients than in controls for different brain regions.

Given the presence of inflammation in Alzheimer's disease, it is reasonable to anticipate an increase in the concentration of TNF-α in the brain. Assuming that the normal brain concentration is comparable to that in cerebrospinal fluid, Csuka et al. (1999) reported a range from 0 to 4.48 pg/mL. To determine the half-maximal rate of neuron death, we consider a TNF-α concentration equal to the maximum value within the normal range. Thus, we set $K_{T_\alpha} = 4.48 \times 10^{-12}$ g/mL.

Some articles provide information on the average or median concentration of IL-10 in CSF for both control individuals and patients with AD. These values are compiled in Table 1. Consensus among most studies indicates a normal concentration below 6 pg/mL. To calculate the average, we exclude the data from Hayakata et al. (2004) and Kirchhoff et al. (2008) as they do not provide an exact value in their studies. We take this average as the concentration of IL-10 at which the rate of neuronal death caused by TNF-α is reduced by half, i.e. $K_{I_{10}} = 2.12 \times 10^{-12}$ g/mL.

Source	Controls (pg/mL)	AD (pg/mL)	Note
Jensen et al. (2019)	0.1	-	
Csuka et al. (1999)	0.16	-	Upper normal value: 1.06 pg/mL
Peters et al. (2017)	$0.5^{\dagger}$	-	Expression level in the motor cortex
Stoeck et al. (2005)	$1.3^{\dagger}$	$7.9^{\dagger}$	
Llano et al. (2012)	2.60	2.45	
Hayakata et al. (2004)	< 3.9	-	Below detection limit
Hu et al. (2019)	4.23	5.5	
Kirchhoff et al. (2008)	< 5	-	Below detection limit
Rauchmann et al. (2020)	5.92	5.445	
Rota et al. (2006)	14.2	21.0	
Tarkowski et al. (2001)	22	45	

Table 1. Average concentrations of IL-10 in the CSF, or otherwise specified, found in the literature for controls and AD patients. †, median.

We adopt a sigmoid function in our model. This decision is based on the observation that NFT accumulation precedes neuronal death, as evidenced by images illustrating distinct stages of neurons with NFTs in Braak and Del Tredici (2010, Figure 1) and Moloney et al. (2021, Figure 3). Consequently, a sigmoid function is deemed more suitable than the Michaelis-Menten equation for capturing this relationship.

1.8 Equation for astrocytes

In equation (14), $A_{\max}$ is the maximal density of activated astrocytes. By observing Figure 3 in Pelvig et al. (2008), we note that there is little or no loss of astrocytes with age. Therefore, we assume that $A_{\max}$ is constant and equal to A_0. Thus, we have $A^F_{\max} = 0.10\,\mathrm{g/cm^3}$ and $A^M_{\max} = 0.12\,\mathrm{g/cm^3}$ for females and males, respectively.

The nuclear factor kappa-light-chain-enhancer of activated B cells (NF-κB) is a transcription factor that regulates cytokine production and cell survival (Liddelow and Barres, 2017). It is strongly associated with neuroinflammation and neuroinflammatory reactivity in astrocytes and microglia (Liddelow and Barres, 2017). The activation (nuclear translocation) of NF-κB in astrocytes is believed to be a result of their activation (Lian et al., 2015; Liddelow and Barres, 2017). Russ et al. (2021, figure S5.A) observed the percentage of human induced pluripotent stem cell-derived astrocytes, activated by 100 ng/mL of TNF-α or control, are showing nuclear translocation of NF-κB over time. After 24 h, they found that approximately 95% of TNF-α-activated cells exhibited nuclear translocation of NF-κB, compared to about 3% for the control group. Therefore, 92% of the cells were activated by TNF-α within 24 h. This yields the following rate:

$$\kappa_{T_\alpha A} = 0.92 \left(\frac{1}{100 \times 10^{-9}\,\mathrm{g/mL}} \right) /\mathrm{d} = 9.2 \times 10^6\,\mathrm{mL/(g\,d)}. \tag{72}$$

Astrocyte activation is primarily driven by proinflammatory cytokines, represented by TNF-α, rather than Aβ. This can be observed by comparing Figures 2 and 5 of Zhao et al. (2011), where increased BACE1 production indicates a higher proportion of activated astrocytes. However, the author mentions that astrocytes activated by TNF-α+IFN-γ or Aβ42 both cause similar increases in APP and BACE1 production, despite some differences. Assuming that the difference between

the two activation pathways is moderate, we propose that

$$\kappa_{A\beta_p^o A} A\beta_p^o = \frac{1}{2}\kappa_{T_\alpha A} T_\alpha. \tag{73}$$

We assume this holds for normal concentrations of TNF-α and Aβ plaques. Thus,

$$\kappa_{A\beta_p^o A} = \frac{\kappa_{T_\alpha A} \cdot T_\alpha(0)}{2 \cdot A\beta_p^o(0)}. \tag{74}$$

We defined $K_{A\beta_p^o} = 3.11 \times 10^{-6}\,\text{g/mL}$ as the concentration of amyloid plaques in the gray matter of controls (see section 1.3). Thus, we take the normal concentration of plaques to be $A\beta_p^o(0) = 3.11 \times 10^{-6}\,\text{g/mL}$. Additionally, we found that the range provided by Csuka et al. (1999) for the normal concentration of TNF-α, which is 0 to 4.48 pg/mL, seemed to be suitable for most studies (see section 1.7). Therefore, we consider the normal concentration of TNF-α to be in the middle of this range, $T_\alpha(0) = 2.24 \times 10^{-12}\,\text{g/mL}$. We therefore take

$$\kappa_{A\beta_p^o A} = \frac{\kappa_{T_\alpha A} \cdot T_\alpha(0)}{2 \cdot A\beta_p^o(0)} = \frac{9.2 \times 10^6\,\text{mL/(g d)} \cdot 2.24 \times 10^{-12}\,\text{g/mL}}{2 \cdot 3.11 \times 10^{-6}\,\text{g/mL}} = 3.3136\,\text{mL/(g d)}. \tag{75}$$

As mentioned in the text (section 2.9), we assume that the death rate, d_A, is constant. We assume a value of 40% per day, i.e., $d_A = 0.4/\text{d}$. This means that the total activation in magnitude would increase each day, which is in agreement with the idea of Sofroniew (2020), since the inflammation due to Alzheimer's disease does not resolve.

1.9 Equations for microglia

Resting microglia

Consider the equation (15). We supposed that the sum of activated and non-activated microglia is approximately constant (Pelvig et al., 2008, Figure 3). Therefore, when proinflammatory or anti-inflammatory microglia die or are deactivated, they are replaced by resting microglia. This occurs at rates $d_{M_{pro}}$ and $d_{M_{anti}}$, respectively.

We assume that the deactivation rate of microglia is small compared to their rate of death. From Ru et al. (2017), we get that in the healthy human brain, microglia are replenished at a median rate of 28 %/year and of an average age of 4.2 years. We thus take

$$d_{M_{pro}} = d_{M_{anti}} = 7.67 \times 10^{-4}/\text{d}. \tag{76}$$

Now, consider the parameters in the equation (16). We have $\kappa_{F_o M}$ describing the maximal activation rate of microglia by extracellular NFTs, K_{F_o} the concentration of extracellular NFTs at which this activation rate is half maximal and, similarly, we have $\kappa_{A\beta_o^o M}$ and $K_{A\beta_o^o}$ for the activation by Aβ oligomers.

In the absence of a better hypothesis, we assume that at most 20% of non-activated microglia are activated each day. In other words, when $\frac{F_o}{F_o + K_{F_o}}$ and $\frac{A\beta_o^o}{A\beta_o^o + K_{A\beta_o^o}}$ are approximately equal to 1, we have that the total activation is given by

$$M_{activ} = \left(\kappa_{F_o M} + \kappa_{A\beta_o^o M}\right) M_{NA} = 0.20 \times M_{NA}.$$

We assume that two-thirds of the activation is caused by NFTs and one-third by amyloid. We consider that the activation due to NFTs is more important as in Hao and Friedman (2016). We

thus have

$$\kappa_{F_oM} = \frac{2}{3} \cdot 0.20/\text{d} \approx 0.133/\text{d}, \text{ and} \tag{77}$$

$$\kappa_{A\beta_o^oM} = \frac{1}{3} \cdot 0.20/\text{d} \approx 0.067/\text{d}. \tag{78}$$

Khatoon et al. (1992) measured between 6 and 16 NFTs in a sample of 1 µL of the homogenized grey matter of AD patients. We therefore consider an average of 11 NFTs per 1 µL. We suppose that this quantity corresponds to the extracellular NFT concentration for which the activation of microglia is half maximal. We assume that the molar mass of hyperphosphorylated tau is 72 500 g/mol; as Sjgren et al. (2001) gives the interval 65 to 80 kDa. We also assume that one NFT is an aggregation of 1000 phosphorylated tau proteins. We thus obtain

$$K_{F_o} = 11/\mu\text{L} \cdot 1000\,\frac{\mu\text{L}}{\text{mL}} \cdot \frac{1000 \cdot 72\,500\,\text{g/mol}}{6.02214076 \times 10^{23}/\text{mol}} = 1.3243 \times 10^{-12}\,\text{g/mL}. \tag{79}$$

For $K_{A\beta_o^o}$, we use Roberts et al. (2017, Table 1). For controls, we get

$$[A\beta_o^o]_{\text{ctrl}} = \frac{0.060\,\text{mg}}{527.4\,\text{mL}} \left(\frac{1\,\text{g}}{1000\,\text{mg}}\right) \approx 1.1377 \times 10^{-7}\,\text{g/mL}. \tag{80}$$

We assume that the activation rate depends linearly on the concentration of Aβ oligomers. We thus have

$$K_{A\beta_o^o} = 1.5 \times 10^2 \cdot [A\beta_o^o]_{\text{ctrl}} = 1.7065 \times 10^{-5}\,\text{g/mL}. \tag{81}$$

This equation has the form of a Michaelis-Menten function describing the kinetics of an irreversible reaction with a unique enzymatic catalyst and a substrate. Hence, the higher $K_{A\beta_o^o}$ is, the more the activation rate curve will look linear. That is why we multiply the concentration by 1.5×10^2 to obtain the parameter.

Activated microglia

We now define the parameters in equations (17) and (18).

For the constant $\kappa_{T_\beta M_{pro}}$, we use the value found in Amato and Arnold (2021), $\kappa_{T_\beta M_{pro}} = 4.8/\text{d}$.

The concentration of TGF-β varies a lot from study to study. Most papers (Vawter et al., 1996; Liu et al., 2022; Tarkowski et al., 2002; Chao et al., 1994; Zetterberg et al., 2004) give average values for controls within the normal interval shown in Csuka et al. (1999) which is 11 to 107 pg/mL. There are however some papers finding values beyond this interval being 492.1 pg/mL (Li et al., 2007), 3 ng/mL (Tarkowski et al., 2001) and 12.0 ng/mL (Rota et al., 2006). Many papers compare TGF-β concentration in CSF in controls and AD patient conclude that there is an increase in concentration (Tarkowski et al., 2002; Chao et al., 1994; Zetterberg et al., 2004; Tarkowski et al., 2001; Rota et al., 2006; for a meta-analysis, see Swardfager et al., 2010), except one (Vawter et al., 1996). We will assume that the concentration increases. We reject data outside the interval of Csuka et al. (1999). It seems reasonable to take the concentration at which the conversion rate is half maximal, $K_{T_\beta M}$, as the average of this interval, being $K_{T_\beta M} = 5.9 \times 10^{-11}\,\text{g/mL}$.

We use the paper of Amato and Arnold (2021) to determine the value of $\kappa_{T_\alpha M_{anti}}$, and obtain $\kappa_{T_\alpha M_{anti}} = 4.8/\text{d}$.

As mentioned for the parameter K_{T_α} in section 1.7, we assume that the normal concentration of TNF-α in the brain is on the same order of magnitude as the concentration in the CSF. From Csuka et al. (1999), we get an average concentration of 2.24 pg/mL. We assume that the rate of

microglia conversion from anti- to proinflammatory depends linearly on the concentration of TNF-α. Here we take $K_{T_\alpha M}$ as 2×10^2 times the normal concentration of TNF-α, that is

$$K_{T_\alpha M} = 2 \times 10^2 \cdot 2.24 \times 10^{-12}\,\text{g/mL} = 4.48 \times 10^{-10}\,\text{g/mL}. \tag{82}$$

The parameter β is the environmental ratio of proinflammatory over anti-inflammatory as determined by the relative strength of TNF-α and IL-10. Thus, if $\beta = 1$, we favor no polarization, if $\beta > 1$, we favor proinflammatory polarization, and if $\beta < 1$ we favor anti-inflammatory polarization. We choose not to foster a polarization, we use $\beta = 1$.

In equation (19), we have two parameters to define: $K_{T_\alpha Act}$ and $K_{I_{10}Act}$. We take the normal concentration of TNF-α, given by Csuka et al. (1999), for $K_{T_\alpha Act}$, hence $2.24 \times 10^{-12}\,\text{g/mL}$. Following what we did while discussing the equations for neurons (section 1.7), we get $K_{I_{10}Act} = K_{I_{10}} = 2.12 \times 10^{-12}\,\text{g/mL}$.

1.10 Activated macrophages

We now define the parameters for the equations (20) and (21).

The constant β, and the parameters in ε_{T_α} and $\varepsilon_{I_{10}}$ are the same as for activated microglia, and have been defined in the preceding section 1.9.

According to experiments performed on mice or rats, the reaction peak of microglia occurs approximately 3-4 days after a brain injury, followed by an importation of blood macrophages 6 to 7 days after trauma (Semple et al., 2010; Hughes et al., 2002; Schroeter et al., 1997; Fumagalli et al., 2015). We assume that macrophage import under MCP-1 signaling takes a minimum of 3 days. We can thus estimate the maximal activation rate by

$$\frac{1}{3\,\text{d}} \approx 3.33 \times 10^{-1}/\text{d}. \tag{83}$$

The activation is slower for macrophages than for microglia and the maximal rate for microglia is 0.20/d (20%/d), with $\kappa_{F_oM} = 0.133/\text{d}$ and $\kappa_{A\beta^o_oM} = 0.067/\text{d}$ (see section 1.9). We divide the rate, of equation (83), by 10^2, so it is inferior to the smallest rate defined for microglia. We obtain $\kappa_{P\hat{M}} = 3.33 \times 10^{-3}/\text{d}$.

We considered 10 studies for the average or median concentration of MCP-1 in the CSF of controls (Galimberti et al., 2006; Jeppsson et al., 2019; Johansson et al., 2017; Kuratsu et al., 1993; Magdalinou et al., 2015; Martnez et al., 2020; Mattsson et al., 2011; Rosn et al., 2014; Santaella et al., 2020; Westin et al., 2012). Two of these studies give a median concentration rather than an average: Martnez et al. (2020); Rosn et al. (2014). The value obtained by Martnez et al. (2020) is relatively smaller than the values given in other studies. They obtain 160.95 pg/mL and the next smallest value is 365 pg/mL. However, the median value given by Rosn et al. (2014) (569 pg/mL) is consistent with the values given in other studies. Generally speaking, we obtain normal concentrations ranging from 160.95 to 860.79 pg/mL, with an average of 500 pg/mL and a median of 454 pg/mL. Six of the papers give either the average or median of MCP-1 concentration in CSF of AD patients (Galimberti et al., 2006; Jeppsson et al., 2019; Magdalinou et al., 2015; Mattsson et al., 2011; Rosn et al., 2014; Westin et al., 2012), and one for those with cognitive impairment (Johansson et al., 2017). The values range from 436 to 1129.04 pg/mL, with an average of 623 pg/mL and a median of 514 pg/mL. Most of the papers observe an increase in MCP-1 concentration in AD patients compared to controls.

We assume that the importation rate depends linearly on the MCP-1 concentration. Thus we take K_P as 10^2 times the average MCP-1 concentration in diseased patients, that is $K_P = 6.23 \times 10^{-8}\,\text{g/mL}$.

We assume that all macrophages can become activated. Most of the proteins expressed by microglia are also expressed by macrophages such as the cluster of differentiation (CD) 68 (CD68) (Hopperton et al., 2018). However, some molecules allow us to distinguish between the

two cells, for instance CD163 (Borda et al., 2008). In Serrano-Pozo et al. (2013), the authors quantify the number of microglia expressing the ionized calcium-binding adapter molecule 1 (IBA1) and the major histocompatibility complex of class II (MHCII or MHC2) in brain samples of controls and AD patients.

The protein IBA1 is expressed in resting microglia and to a larger degree in activated microglia (Serrano-Pozo et al., 2013). MHC2 is expressed by activated microglia and by mononuclear blood cells such as monocytes, lymphocytes, and perivascular macrophages (Serrano-Pozo et al., 2013). It follows that cells not expressing IBA1 but expressing MHC2 are macrophages from the periphery that have been recruited in the brain (Serrano-Pozo et al., 2013). In Serrano-Pozo et al. (2013, Figure 6G), the authors measure a maximum of approximately 830 microglia $IBA1^{-}MHC2^{+}$ in AD patients in a sample of 1 cm long and 8 µm wide with a thickness equal to the full width of the temporal cortex. From Figure 9A of this paper, we assume an average width of 2500 µm for the temporal cortex. This gives a sample volume of

$$V_{\text{sample}} = 1\,\text{cm} \cdot 8 \times 10^{-4}\,\text{cm} \cdot 0.25\,\text{cm} = 2 \times 10^{-4}\,\text{cm}^3 = 2 \times 10^{-4}\,\text{mL}. \tag{84}$$

The volume of a macrophage is approximately $4990\,\mu\text{m}^3 = 4.990 \times 10^{-9}\,\text{mL}$ (Krombach et al., 1997). Assuming they have the same density as water, we get that the mass of a macrophage is

$$m_{\hat{M}} = 4.990 \times 10^{-9}\,\text{g}. \tag{85}$$

Thus, in AD patients, we obtain a maximal macrophage concentration of

$$\hat{M}_{max} = \frac{830 \cdot 4.990 \times 10^{-9}\,\text{g}}{2 \times 10^{-4}\,\text{mL}} \approx 2.071 \times 10^{-2}\,\text{g/mL}, \tag{86}$$

Remark that CD68 production has been used by Pelvig et al. (2008) to discriminate microglia from other cells. As mentioned earlier, this is also a marker of infiltrated macrophages, is it possible that they get a count which is slightly too high? According to Serrano-Pozo et al. (2013, figure 6) only approximately 0.67% of microglia would be $IBA1^{-}MHC2^{+}$ microglia, i.e. macrophages, for controls, as opposed to 6.77% for AD patients. As Pelvig et al. (2008) only considers controls, it is reasonable to believe that the proportion of counted macrophages is negligible.

Since the polarization of macrophages is influenced by the same cytokines as microglia (Martinez and Gordon, 2014; Wang et al., 2021; Orihuela et al., 2016), we take the same equations and same parameters as for polarization of microglia (section 1.9).

In Khallou-Laschet et al. (2010), the authors observe a phenotype switch (pro to anti-inflammatory) in less than 10 h in mice macrophages. We assume that the phenotype switch occurs in 10 h in human macrophages. We thus take the following maximal rate of conversion:

$$\kappa_{T_\beta \hat{M}_{pro}} = \frac{1}{10\,\text{h} \cdot \frac{1\,\text{d}}{24\,\text{h}}} = 2.4/\text{d}. \tag{87}$$

We also take $K_{T_\beta \hat{M}} = K_{T_\beta M} = 5.9 \times 10^{-11}\,\text{g/mL}$ where $K_{T_\beta \hat{M}}$ is the TGF-β concentration for which the conversion of macrophage from pro to anti-inflammatory is half maximal.

In Khallou-Laschet et al. (2010), the authors also observe a phenotype switch (from anti- to proinflammatory) in less than 10 h in mice macrophages. We assume that the phenotype switch occurs in 10 h in human macrophages, we thus take the following maximal rate of conversion:

$$\kappa_{T_\alpha \hat{M}_{anti}} = \frac{1}{10\,\text{h} \cdot \frac{1\,\text{d}}{24\,\text{h}}} = 2.4/\text{d}. \tag{88}$$

We also take $K_{T_\alpha \hat{M}} = K_{T_\alpha M} = 4.48 \times 10^{-10}\,\text{g/mL}$, where $K_{T_\alpha \hat{M}}$ is the TNF-α concentration for which the conversion of macrophage from anti- to proinflammatory is half maximal.

We will consider that the death, or deactivation, rates of macrophages are the same as for microglia (equation (76)). We thus neglect deactivation and use $d_{\hat{M}_{pro}} = d_{\hat{M}_{anti}} = 7.67 \times 10^{-4}$/d (Ru et al., 2017).

1.11 Cytokines and chemokines

TGF-β

For equation (22), Fadok et al. (1998, figure 3) obtained a production rate of TGF-β by macrophages in culture, stimulated by lipopolysaccharides (LPS) and controlled in 18 h, of approximately 47 pg/mL, with 1 to 2 $\times 10^6$ macrophages/well, or per 0.5 mL, since they use a 24-well plate. From this, we can obtain the rate of production by proinflammatory macrophages. We assume that anti-inflammatory macrophages have a ten times greater production rate (Cao et al., 2010, figure 1B). We consider a production rate of

$$10 \cdot \frac{47\,\text{pg/mL}}{18\,\text{h}} \times 24\,\text{h/d} = \frac{1880}{3}\,\text{per-mode=repeated-symbolpg/(mL d)} \approx 626.67\,\text{pg/mL/d}, \tag{89}$$

for a concentration of 2 to 4 $\times 10^6$ macrophages/mL. Using the following approximate value for the mass of a macrophage $m_{\hat{M}} = 4.990 \times 10^{-9}$ g (equation (85)), we have 9.980 to 19.96 $\times$ 10^3 $\text{g}_{\text{macrophage}}$/mL. We thus consider that the value of $\kappa_{\hat{M}_{anti}T_\beta}$ is between

$$\frac{626.67 \times 10^{-12}\,\frac{\text{g}}{\text{mL d}}}{1.996 \times 10^{-2}\,\frac{\text{g}}{\text{mL}}} = 3.14 \times 10^{-8}/\text{d}, \tag{90}$$

and

$$\frac{626.67 \times 10^{-12}\,\frac{\text{g}}{\text{mL d}}}{9.980 \times 10^{-3}\,\frac{\text{g}}{\text{mL}}} = 6.28 \times 10^{-8}/\text{d}. \tag{91}$$

For the sake of simplicity, we assume that the rate of production by microglia is the same as the one by macrophages. We thus have $\kappa_{M_{anti}T_\beta} = 3.14$ to 6.28×10^{-8}/d.

The half-life of the active form of TGF-β in plasma is between 2 and 3 minutes (Wakefield et al., 1990). Using the value of 3 minutes for the brain, we get

$$d_{T_\beta} = \frac{\ln 2}{3\,\text{min} \times \frac{1}{1440}\,\text{d/min}} = 3.33 \times 10^2/\text{d}. \tag{92}$$

To compensate the rapid degradation rate, we use the highest production rates, i.e. $\kappa_{\hat{M}_{anti}T_\beta} = \kappa_{M_{anti}T_\beta} = 6.28 \times 10^{-8}$/d.

IL-10

Consider equation (23). In Mia et al. (2014), the authors stimulate 2×10^5 monocytes/mL with a combination of IL-4, IL-10 and TGF-β and find a production rate of approximately 660 pg/mL en 24 h. We consider that the mass of a monocyte is equal to the mass of a macrophage $m_{\hat{M}} = 4.990 \times 10^{-9}$ g (equation (85)). The rate of production of IL-10 is thus $\kappa_{\hat{M}_{anti}I_{10}} \approx 6.61 \times 10^{-7}$/d.

We assume that the rate is the same for microglia, as was done in Hao and Friedman (2016), that is $\kappa_{M_{anti}I_{10}} = \kappa_{\hat{M}_{anti}I_{10}} = 6.61 \times 10^{-7}$/d.

Regarding the degradation, Huhn et al. (1997) observe that blood IL-10 has a half-life of 3.556 h, in healthy humans of an average age of 25.9 years. We thus have $d_{I_{10}} = 4.6782$/d.

TNF-α

Consider equation (24). In Fadok et al. (1998, figure 3), the authors obtain a production rate of TNF-α by macrophages in culture stimulated by LPS and controled in 18 h of approximately 1.5 ng/mL, with $1to26 \times 10^6$ macrophages/puit, or for 0.5 mL, since they use a 24-well plate. This gives us the production rate by proinflammatory macrophages. The mass of a macrophage was set as $m_{\hat{M}} = 4.990 \times 10^{-9}$ g (equation (85)). With this, we obtain a value of $\kappa_{\hat{M}_{pro}T_\alpha}$ between

$$\frac{\frac{1.5 \times 10^{-9}\,\mathrm{g/mL}}{18\,\mathrm{h}} \times 24\,\mathrm{h/d}}{1.996 \times 10^{-2}\,\frac{\mathrm{g}}{\mathrm{mL}}} = 1.002 \times 10^{-7}/\mathrm{d}, \tag{93}$$

and

$$\frac{\frac{1.5 \times 10^{-9}\,\mathrm{g/mL}}{18\,\mathrm{h}} \times 24\,\mathrm{h/d}}{9.980 \times 10^{-3}\,\frac{\mathrm{g}}{\mathrm{mL}}} = 2.004 \times 10^{-7}/\mathrm{d}. \tag{94}$$

We assume that this rate is the same for microglia, that is $\kappa_{M_{pro}T_\alpha} = (1,002 \text{ to } 2,004) \times 10^{-7}/\mathrm{d}$.

The half life of TNF-α in plasma is 18.2 min (Oliver et al., 1993), in agreement with Zahn and Greischel (1989). We thus use $d_{T_\alpha} = 54.84/\mathrm{d}$.

As the degradation rate is smaller than the one for TGF-β, which is $3.33 \times 10^2/\mathrm{d}$, we used the smaller production value in order to minimize the difference between equilibrium concentrations, for equal concentrations of pro and anti-inflammatory microglia and macrophages. Thus, $\kappa_{\hat{M}_{anti}T_\alpha} = \kappa_{M_{pro}T_\alpha} = 1.002 \times 10^{-7}/\mathrm{d}$.

MCP-1

Consider equation (25). Yoshimura et al. (2016) measure the production of MCP-1 for peritoneal exudate cells of wild-type mice which were induced by intraperitoneal injection of thioglycolate at 3% and harvested 3-4 days later. The peritoneal exudate cells are neutrophilic leukocytes, macrophages and lymphocytes. Peritoneal exudate is usually collected to obtain leukocytes, including free macrophages. From Figure 4C of this article, we learn that two millions (2×10^6 cells/mL) of these cells (macrophages) activated by LPS, hence of proinflammatory polarization, produce approximately 11 ng/mL of MCP-1 in 24 h. We fixed the mass of a macrophage to $m_{\hat{M}} = 4.990 \times 10^{-9}$ g (see equation (85)). Therefore, that production is for 9.980×10^{-3} g/mL of macrophages. Hence, we have

$$\kappa_{\hat{M}_{pro}P} = \frac{11 \times 10^{-9}\,\mathrm{g/mL/d}}{9.980 \times 10^{-3}\,\mathrm{g/mL}} \approx 1.1 \times 10^{-6}/\mathrm{d}. \tag{95}$$

We assume that the rate of production of MCP-1 by microglia is the same as the rate of production by macrophages, i.e. $\kappa_{M_{pro}P} = 1.1 \times 10^{-6}/\mathrm{d}$.

As mentioned, monocytes and macrophages are the main source of MCP-1 (Lee et al., 2018). We assume that the rate of production by astrocytes is 10 times smaller than the rate of production by microglia/macrophages $\kappa_{AP} = 1.1 \times 10^{-7}/\mathrm{d}$.

From Cheng et al. (2005), the half-life of MCP-1 in mesangial cells of rats is approximately 3 h. We assume that this is equal to the half-life in the human brain. We thus get $d_P = 5.5452/\mathrm{d}$.
